# Fluorescent Sensing of SO_2_ by MFM‐300(M) Metal–Organic Frameworks: Influence of Semi‐Open Metal Centres

**DOI:** 10.1002/smll.202507448

**Published:** 2025-09-01

**Authors:** Valeria B. López‐Cervantes, Hashim Alhashimi, Christian A. Celaya, M. Solórzano, Marco L. Martínez, Yoarhy A. Amador‐Sánchez, Evandro Castaldelli, Edward Lester, Ricardo A. Peralta, Enrique Lima, Diego Solis‐Ibarra, Sihai Yang, Ilich A. Ibarra, Andrea Laybourn

**Affiliations:** ^1^ Laboratorio de Fisicoquímica y Reactividad de Superficies (LaFReS) Instituto de Investigaciones en Materiales Universidad Nacional Autónoma de México Circuito Exterior s/n, CU, Coyoacán Ciudad de México 04510 México; ^2^ Advanced Materials Research Group Faculty of Engineering University of Nottingham Nottingham NG7 2NR UK; ^3^ Centro de Nanociencias y Nanotecnología Universidad Nacional Autónoma de México Km 107 Carretera Tijuana‐Ensenada Ensenada Baja California 22860 México; ^4^ Laboratorio de Investigación en Materiales Porosos Catálisis Ambiental y Química Fina ESIQIE – Instituto Politécnico Nacional Avenida IPN UPALM Edificio 7, Zacatenco Ciudad de México 07738 México; ^5^ Departamento de Química Universidad Autónoma Metropolitana‐Iztapalapa Av. Ferrocarril San Rafael Atlixco 186, Col. Leyes de Reforma 1A Sección, Iztapalapa Ciudad de México 14387 México; ^6^ College of Chemistry and Molecular Engineering Beijing National Laboratory for Molecular Sciences Peking University Beijing 100871 China; ^7^ Institute of Process Research and Development & School of Chemistry University of Leeds Leeds LS2 9JT UK

**Keywords:** fluorescent sensing, metal‐organic frameworks, MFM‐300, sulfur dioxide

## Abstract

The MFM‐300(M) series (M = Al(III), Sc(III), Cr(III), and In(III)) have previously demonstrated excellent sulfur dioxide (SO_2_) adsorption capabilities, however, their potential as fluorescent SO_2_ sensors remains unexplored. Here, this work presents a comparative study of their fluorescence response upon SO_2_ exposure, with a particular focus on the role of the metal centers. MFM‐300(Al) exhibits the strongest emission and highest quenching upon SO_2_ exposure, attributed to localized interactions with *µ*_2_‐OH functional groups and aromatic sites. In contrast, Sc(III) and In(III) analogues show moderate quenching via charge transfer at the semi‐open metal sites, while Cr(III) remains weakly emissive. Density Functional Theory (DFT) calculations employing periodic boundary conditions are conducted to characterize the electronic structure of MFM‐300(M), to elucidate the role of metal centers in SO_2_ retention and to assess the semiconducting nature of these metal‐ organic frameworks (MOFs).

## Graphical abstract

The MFM‐300(M) series is investigated as a fluorescent platform for SO_2_ detection, highlighting how the nature of metal centers modulates fluorescence emission. MFM‐300(Al) displays the brightest emission and strongest response. Experimental and computational studies reveal distinct quenching mechanisms depending on the metal center, highlighting the role of static versus dynamic host‐guest interactions in tuning fluorescence response.

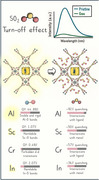

## 1 Introduction

Sulphur dioxide (SO_2_) is a colorless, pungent‐smelling gas that is mainly generated anthropogenically by the burning of fossil fuels, such as coal and oil, and by the smelting of sulphur‐containing ores.^[^ ^1^ ^]^ SO_2_ emissions contribute significantly to air pollution, a global problem affecting both urban and rural areas.^[^ ^2^ ^]^ It has also been listed by the World Health Organisation as one of six pollutants critical to human health.^[^ ^3^ ^]^ The exposure to low concentrations of SO_2_ (5–8 ppm) mainly affect the respiratory system, causing respiratory tract irritation, coughing and shortness of breath.^[^ ^4^ ^]^ In people with pre‐existing respiratory diseases, such as asthma, exposure to SO_2_ can trigger severe attacks.^[^ ^5^ ^]^ In addition, SO_2_ can cause severe cases of dermatitis and conjunctivitis,^[^ ^6^, ^7^ ^]^ as well as aggravate cardiovascular disease,^[^ ^8^ ^]^ increasing the risk of heart attacks and other serious health problems. From an environmental point of view, SO_2_ is a key component in the formation of acid rain:^[^ ^9^ ^]^ when SO_2_ is released into the atmosphere, it can react with water and oxygen, as well as interact with ultraviolet (UV) radiation,^[^ ^10^ ^]^ leading to the formation of its acidic derivatives, such as sulfuric acid. The resulting acid rain can have devastating effects, damaging forests, soils and water bodies,^[^ ^11^ ^]^ adversely affecting flora and fauna,^[^ ^12^ ^]^ and even causing damage to metal and non‐metal surfaces of buildings.^[^ ^13^ ^]^ In addition, SO_2_ contributes to the formation of sulphate aerosols (SO_4_^2−^),^[^ ^14^ ^]^ the main components of fine particulate matter (PM_2.5_) which act as cloud condensation nuclei, altering the optical and radiative properties of clouds, leading to the scattering of solar radiation, thus affecting the climate,^[^ ^15^ ^]^ and can also penetrate deep into the lungs and cause serious health problems. Therefore, it is essential to develop effective methods for SO_2_ detection and monitoring to keep “SO_2_‐safety” in different industries and factories where it is necessary to protect the health of personnel exposed to this toxic gas, as well as for optimizing operational processes by identifying and correcting any inefficiencies or problems in production that may be causing unnecessary emissions, thus reducing the environmental impact. Currently, most commercial SO_2_ detectors are based on semiconduction and electrochemistry, with a lifetime of between 24 and 36 months. In semiconductor‐based detectors SO_2_ reacts with the detector surface, causing the SO_2_ gas molecules to dissociate into charged ions that alter the resistance of the film; this interaction is measured as an electrical signal whereby intensity depends on the gas concentration.^[^ ^16^ ^]^ However, these detectors have limitations such as low selectivity.^[^ ^17^ ^]^ In the case of electrochemical detectors, after SO_2_ diffuses into the device, it can be oxidized or reduced at the working electrode, which generates an electric current that is proportional to the concentration of SO_2_.^[^ ^18^ ^]^ While electrochemical sensors offer advantages such as sensitivity and low cost,^[^ ^19^ ^]^ their regular calibration and maintenance required to ensure accuracy over time results in high operational and maintenance costs.^[^ ^20^ ^]^

An alternative for SO_2_ detection is the fluorescence approach, which takes advantage of the fluorescent properties of certain materials that, when interacting with guest molecules, change either the intensity or the shape of the detector's fluorescence emission spectrum, allowing molecule quantification. Commonly, fluorescent detectors are based on traditional organic and polymeric fluorophores.^[^ ^21^ ^]^ However, an emerging option is the use of metal‐organic frameworks (MOFs).^[^ ^22^ ^]^ MOFs are a class of hybrid materials constructed from metal nodes/clusters and organic ligands that, via coordination bonds, form highly porous and crystalline structures. These structures offer materials with high surface areas and preferential adsorption sites which can selectively interact with SO_2_.^[^ ^23^ ^]^ In addition, their structures contain π and n electrons, which are conducive to the formation of variable fluorescence signals.^[^ ^22^, ^24^ ^]^

**Figure 1:**
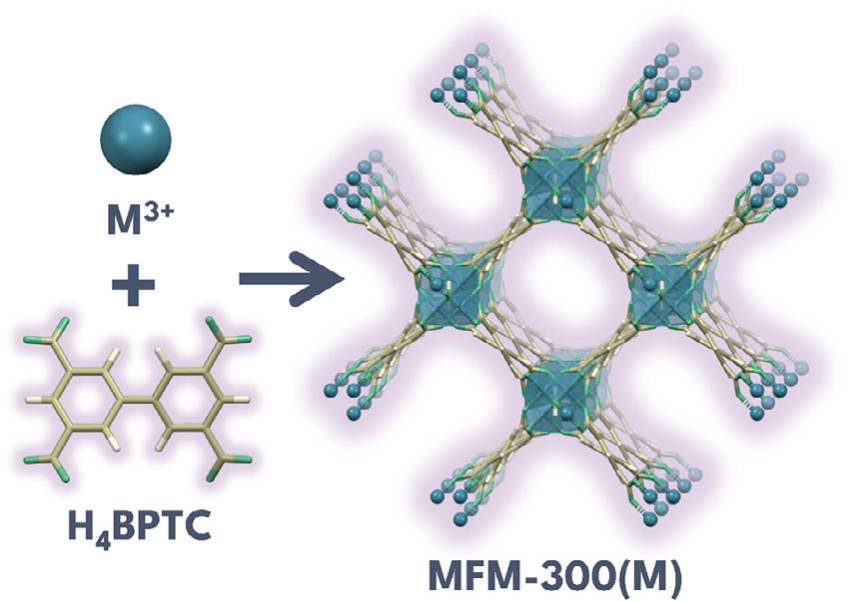
Structure of MFM‐300(M) family.

MFM‐300(M) is a MOF series constructed from trivalent metal centers such as Al(III),^[^ ^25^ ^]^ Sc(III),^[^ ^26^ ^]^ Cr(III),^[^ ^27^ ^]^ and In(III),^[^ ^28^ ^]^ and biphenyl‐3,3′,5,5′‐tetracarboxylic acid (H_4_BPTC) ligand, which form octahedral clusters [MO_4_(OH)_2_], where the metal centers are coordinated to four O‐atoms in axial positions from four different carboxylate groups, and to two O‐atoms in equatorial positions that form *µ_2_*‐OH bridging groups (**Figure** **1**), coordinating two metal atoms to each other in *cis* position. The octahedral secondary building units (SBUs) [MO_4_(OH)_2_] are connected via BPTC⁴^−^ ligands, forming interconnected helical chains in a 3D arrangement. The resulting 3D structure forms square‐shaped channels containing the *µ_2_*‐OH ligands pointing toward the center of the pore, creating a specific chemical environment within the channel. It is worth mentioning that the *cis‐µ_2_*‐OH arrangement observed in the MFM‐300(M) series, provides a rigid framework for the 1D channels, conversely to the *trans‐µ_2_*‐OH arrangement observed in the MIL‐53 series which imparts flexible properties to the framework.^[^ ^29^ ^]^ Finally, the whole MFM‐300(M) series crystallizes in the tetragonal space group *I4_1_22*.

**Table** **1** summarizes the principal characteristics of some of the most representative members of the MFM‐300(M) series. The potential of these MFM‐300(M) (M = Al(III), Sc(III), Cr(III) and In(III)) materials for SO_2_ capture has been previously explored, showing promising results since these materials are highly chemical stable (i.e., robust) toward SO_2_ (anhydrous) and even under wet conditions. The number of adsorbed molecules per unit cell in each MFM‐300(M) at 1 bar is presented in Table 1 with supporting calculations are provided in Section S2.2.

**Table 1:** Textural and SO_2_ adsorption properties of MFM‐300(M) series.

Material	Surface area [m^2^ g^−1^]	Pore volume [cm^3^ g^−1^]	Pore size [Å]	SO_2_ uptake [mmol g^−1^]	SO_2_ molecules per unit cell	Enthalpy of adsorption [kJ mol^−1^]	Ref.
MFM‐300(Al)	1370	0.38	7.5^a^	8.1	3.29	27–30	[25]
MFM‐300(Cr)	1045	0.47	7.5^b^	8.6	3.59	39.3	[27]
MFM‐300(Sc)	1350	0.56	8.1^c^	9.4	4.23	36.2	[26]
MFM‐300(In)	1065	0.37	7.6^d^	8.3	4.89	34.5	[28]

Pore size determined by ^a)^Monte Carlo/DFT simulation, ^b)^structural analysis based on X‐ray diffraction with synchrotron radiation, ^c)^Dubininin‐Astakhov (DA) analysis applied to N_2_ adsorption isotherm at 77 K and ^d)^Horváth‐Kawazoe (HK) method applied to N_2_ adsorption isotherm at 77 K.

Interestingly, MFM‐300(Sc) and MFM300(In) demonstrate dynamic metal‐ligand bonding properties, under different guest molecules, which provides access to temporary open metal sites (semi‐open metal sites), affording unexpected catalytic properties.^[^ ^30^ ^]^ In MFM‐300(Sc) and MFM300(In), such dynamics occur through reversible reconfiguration of the bonds between the metal center and carboxylate ligands in response to the inclusion of certain host molecules. This structural flexibility allows certain adsorbates, such as ammonia (NH_3_),^[^ ^31^, ^32^ ^]^ to temporarily alter the coordination geometry of the metal center (i.e., Sc(III)), facilitating the formation of reversible open metal sites where capture can occur. This host‐structure interaction is not simply a static adsorption process; it involves a dynamic equilibrium that arises due to the hemilability of the metal‐ligand bonds.^[^ ^33^ ^]^ This means that, rather than being static bonds, one of the ligand donor O‐atoms can reversibly de‐coordinate from the metal center, allowing the temporary creation of open metal sites, facilitating stronger or more specific interactions with the adsorbates and, at the same time, allowing the bond to regenerate to restore the original structure of the material (i.e., dynamic bonds).Thus, these semi‐open metal sites, within MFM‐300(Sc) and MFM‐300(In), can strongly interact with guest molecules (e.g., SO_2_), affording new and exciting applications of this promising series of MOF materials (MFM‐300(M)).

In this work, motivated by the previously reported results on the successful SO_2_ capture in MFM‐300(M) (M = Al(III), Sc(III), Cr(III) and In(III)) and the uncharacteristic dynamic metal‐ligand bonding phenomenon, observed in two members of this series, we investigated the SO_2_ detection properties of the MFM‐300(M) (M = Al(III), Sc(III), Cr(III) and In(III)) series.

## 2 Results and Discussion

### 2.1 MFM‐300(M) Characterization

MFM‐300(M) MOFs were synthesised as reported in the literature,^[^ ^26^, ^34^, ^35^, ^36^ ^]^ see Section S1.2 of the Supporting Information (SI). A solvent exchange with acetone promotes easier desolvation and subsequent activation when the materials are placed under vacuum, then activated at 150 °C under vacuum. Phase purity of the as‐synthesised materials was corroborated by powder X‐ray diffraction (PXRD) after solvent exchange (Figure S2, Supporting Information). Fourier‐transform infrared (FTIR) spectra of the MFM‐300(M) materials are presented in Figure S5, Supporting Information. The spectrum of MFM‐300(Al) (Figure S5, Supporting Information), showed strong bands at 1394 cm^−1^ attributable to the symmetric vibration of the carboxylate ν_s_(C═O), and at 1652 cm^−1^ assigned to the asymmetric stretching vibration of the carboxylate ν_as_(C═O).^[^ ^37^ ^]^ A small peak is also observed at 3692 cm^−1^ which is associated with the vibration of the O─H bond of the µ‐OH framework (Figure S6, Supporting Information).^[^ ^38^ ^]^ Finally, peaks are observed at 1582 and 1442 cm^−1^ corresponding to the C─C stretch (in‐ring), and at 1256 and 1099 cm^−1^ of the asymmetric and symmetric deformation modes of the C─H bond, respectively.^[^ ^31^ ^]^

### 2.2 MFM‐300(M) Fluorescence

**Figure 2:**
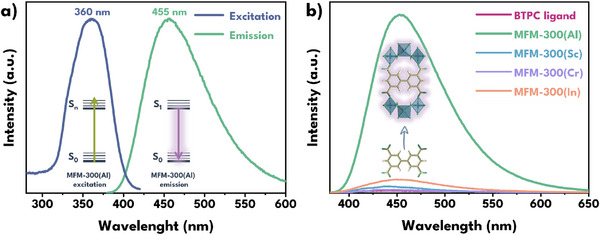
a) Solid‐state fluorescence excitation (blue) and emission (green) spectra of MFM‐300(Al) material. b) Solid‐state fluorescence emission spectra of MFM‐300(M) materials and free ligand H_4_BPTC (λ_ex_ = 360 nm).

Absorption spectra of the activated Al(III), Sc(III), Cr(III) and In(III) MFM‐300 materials showed that photon absorption in these materials occurs in the region ≈200–350 nm (Figure S7, Supporting Information), which is characteristic of π→π* transitions in the aromatic ligand system. The solid‐state emission spectra of MFM‐300(M) materials and H_4_BPTC ligand at λ_ex_ = 360 nm were measured (**Figures** **2** and S10, Supporting Information). The ligand exhibits a moderate emission in the 420 to 550 nm region, with a quantum yield (QY) of 0.31% while the MFM‐300(M) materials exhibit slightly variable emission behavior depending on the metal center constituting the structure. MFM‐300(Al) stands out for its strong emission centered ≈450 nm with a QY of 44.89%, compared to the Sc(III), Cr(III), In(III) analogues, which exhibit QY values of 1.07, 0.49, and 2.38%, respectively.

Although H_4_BPTC has an extended conjugated structure, which allows the absorption and emission of light in the ultraviolet‐visible region of the spectrum (Figure 2), the presence of carboxylic groups with a negative inductive effect (‐I) generates a redistribution of the electron density of the aromatic rings to which they are attached.^[^ ^39^ ^]^ By removing electron density from the biphenyl conjugate system, the π electrons may become less available for fluorescence, making non‐radiative transitions more probable.^[^ ^40^ ^]^ In fact, these groups (─COOH) have been observed to favor intramolecular photoinduced electron transfer.^[^ ^41^, ^42^ ^]^ Coordination of H_4_BPTC with metals has been reported to modify the twist between the benzene rings, leading to alteration of the fluorescent properties of the ligand.^[^ ^43^, ^44^ ^]^ In this work, we observed that coordination with different trivalent metals ions enhances the emission of BPTC^4−^, without significantly changing the emission wavelength, suggesting that the origin of fluorescence in the MFM‐300(M) series is ligand‐centered and that the metal center can modulate the emission intensity.^[^ ^45^ ^]^ In general, the increase in fluorescence after ligand and metal coordination can be associated with reduced dynamics within the structure.^[^ ^46^ ^]^

There are several materials with outstanding fluorescent properties that are constructed with Al(III) metal centers.^[^ ^47^, ^48^ ^]^ A remarkable case is aluminium tris(8‐hydroxyquinolinate) (Alq_3_),^[^ ^49^ ^]^ a compound in which Al(III) coordinates with three 8‐hydroxyquinolinate ligands, forming a distorted octahedral structure. This compound is widely used in organic light‐emitting diodes (OLEDs) due to its ability to transport electrons and its emission efficiency in the green‐yellow region of the visible electromagnetic spectrum.^[^ ^50^ ^]^ In this case, as in our study, the fluorescence is mainly due to π‐π* transitions within the ligands; however, the presence of Al(III) as a coordination metal center stabilizes the molecular structure and favorably modulates the electronic environment. Menglin and co‐workers studied fifty‐five 8‐hydroxyquinoline (8‐HQ)‐derived compounds synthesised with Al(III), Cd(II), Cu(II), and Zn(II), observing that in general, Al(III) complexes showed stronger fluorescence emission intensity suggesting that it influences the electronic distribution of the system by modulating the molecular energy levels due to differences in the coordination and steric effect of the metal.^[^ ^51^ ^]^

**Figure 3:**
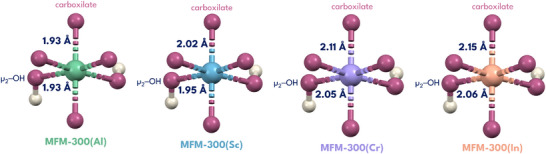
Metal coordination environments in the MFM‐300(M) series (M = Al, Sc, Cr, In), showing M─O bond distances to carboxylate and *µ_2_*‐OH. Colour code: O (pink), H (white), Al (green), Sc (blue), Cr (purple), and In (orange).

With the above in mind, when comparing the ionic radii of each metal in the MFM‐300(M) series: Al(III) ≈0.50 Å, Sc(III) ≈0.75 Å, Cr(III) ≈0.62 Å, and In(III) ≈0.80 Å, Al(III) shows the smallest ionic radius, which indicates it has the highest charge density.^[^ ^52^ ^]^ This makes it the most polarizing cation in the series, leading to metal‐ligand interactions with greater covalent character.^[^ ^53^ ^]^ The Al(III)─O_ligand_ bond, is the shortest and most stable M(III)─O bond in the MFM‐300(M) series, as shown in **Figure** **3**. By correlating the M─O bond length, specifically, the distance between the metal center and the oxygen atom of the ligand, to the dynamic metal‐ligand bonding properties of the MFM‐300(M) series, it can be proposed that MFM‐300(Al) exhibits the least dynamic behavior, which can be translated into a disfavoring of the non‐radiative mechanisms that may complement the dynamics of the framework. Maurin et al., computationally proposed that MFM‐300(Al) does not exhibit dynamic bonds under the presence of NH_3_,^[^ ^31^ ^]^ which supports our hypothesis that from the four investigated materials, MFM‐300(Al) is the least dynamic.

The fluorescence decay spectra at λ_em_ = 455 nm of the H_4_BPTC ligand and MFM‐300(M) materials in the solid state (Figure S16, Supporting Information) exhibit a multiexponential character that fits well with three components on the nanosecond scale (Table S6, Supporting Information). For the ligand, the average lifetime ⟨τ⟩ is 3.27 ns, resulting from a short component (τ_1_ ≈ 0.57 ns, a_1_ ≈ 29%), an intermediate one (τ_2_ ≈ 2.32 ns, a_2_ ≈ 48%) and a long one (τ_3_ ≈ 8.62 ns, a_3_ ≈ 23%), indicating that fast and slow deactivation processes coexist, probably related to the internal mobility of the chromophore and its packing in the solid state. For MFM‐300(Al), ⟨τ⟩ increases to 3.79 ns, with a marked reduction in the contribution of the fast fraction (τ_1_ ≈ 1.00 ns, a_1_ ≈ 14%) and an increase in the intermediate component (τ_2_ ≈ 3.40 ns, a2 ≈ 59%), while the long component remains almost unchanged (τ_3_ ≈ 6.20 ns, a_3_ ≈ 26%). This temporal redistribution in which the intermediate fraction gains strength, together with the high quantum yield (44.89%), reinforces the idea that coordination to Al^3+^ restricts internal movements of the free ligand that facilitate ultrafast non‐radiative deactivations.

MFM‐300(Sc) shows ⟨τ⟩ of 2.73 ns with a_1_ ≈ 37%, a_2_ ≈ 40% and a_3_ ≈ 23%, indicating a more balanced competition between the fast and slow pathways, which is consistent with its QY of 1.07%. Something similar happens with MFM‐300(In), which has ⟨τ⟩ of 2.45 ns with a_1_ ≈ 44%, a_2_ ≈ 35% and a_3_ ≈ 21% with a QY of 2.38%, suggesting that the suppression of the fast mechanisms is also partial. MFM‐300(Cr) shows slightly different behavior, with a reduction in ⟨τ⟩ to 1.42 ns, with its fast fraction dominant (a_1_ ≈ 56%), indicating high non‐radiative deactivation efficiency, in agreement with its extremely low QY (0.49%) related to its 3d^3^ configuration, which introduces non‐radiative d‐d transitions. Overall, the Time‐resolved photoluminescence (TRPL) experiments on pristine materials show that the increase in the long τ_2_ and τ_3_ contributions correlates with higher quantum efficiencies, while the decrease in the contribution of the short τ_1_ component corroborates the idea of a decrease in non‐radiative mechanisms after framework formation.

In the MFM‐300(M) series, it could be hypothesized that the shortest lifetime component (τ_1_) is related to the aromatic moiety of the ligand, since studies in small molecular systems show that aromatic groups with a certain degree of torsionality can have short deactivation times, which would be associated with non‐radiative losses associated with mechanical movements of the conjugated π structure. Furthermore, according to Baird's rule, a system that exhibits aromaticity in the ground state can become antiaromatic in the first singlet excited state (S_1_), and vice versa.^[^ ^54^ ^]^ Thus, Kotani and coworkers demonstrated that when the system has an antiaromatic character in the S_1_ state, it becomes destabilized and tends to relax rapidly through non‐radiative pathways, which results in a shortening of the fluorescence lifetime.^[^ ^55^ ^]^ Since the BPTC^4‐^ ligand contains two aromatic benzene rings, this hypothesis could be applicable, suggesting that τ_1_ in MFM‐300(M) partly reflects the rapid relaxation associated with antiaromatic destabilization in S_1_. Thus, the variation in τ_1_ after coordination with the metal centers could reflect differences in the degree of conformational restriction of the ligand when building the MFM‐300(M) series. For example, it would corroborate that the framework constructed from Al(III) is the least dynamic, as it shows the slowest τ_1_ component and the lowest contribution of the entire series.

In order to evaluate the spectral contribution of each lifetime component (τ_1_, τ_2_ and τ_3_), solid‐state decay spectra were recorded in the range of 400 to 600 nm, accumulating data for 10 s at each wavelength. From this data, a global multiexponential fit was performed, from which the amplitudes of each component at each wavelength were obtained. Decay‐Associated Spectra (DAS) for the H_4_BPTC ligand and the MFM‐300(M) materials (Figures S20 to S24, Supporting Information) were obtained, in order to provide a spectral representation of each lifetime component obtained in a global time‐resolved fluorescence analysis, thus facilitating the interpretation of relaxation and energy transfer mechanisms.^[^ ^56^ ^]^ In the case of the ligand, it can be seen that τ_1_ dominates over spectral range, indicating that rapid deactivation processes are widely distributed in the emission spectrum; on the other hand, the contributions of τ_2_ and τ_3_ throughout the spectrum are considerably smaller and practically flat, suggesting that in the free ligand, the emission is strongly conditioned by ultrafast non‐radiative loss processes. In contrast, in MFM‐300(Al), the amplitude of τ_1_ decreases dramatically, concentrating in the blue region (410 nm), consistent with the increase in average lifetime and quantum yield in this material due to the suppression of fast processes. τ_2_ and τ_3_ maintain a uniform and weak presence throughout the spectrum. Meanwhile, in MFM‐300(Sc) and MFM‐300(In), τ_1_ has a strong presence (even more than in the ligand), especially in the blue and red parts of the spectrum, which matches the lower QY values compared to the Al(III) analogue. Then, MFM‐300(Cr) shows a pattern dominated by τ_1_ across virtually the entire spectrum, with amplitudes similar to those of the ligand and minimal contributions from τ_2_ and τ_3_, suggesting that long‐lasting fluorescence is strongly suppressed.

To further explore the influence of metal‐ligand coordination on the electronic properties of the materials, we compared the Highest Occupied Molecular Orbital (HOMO)‐Lowest Unoccupied Molecular Orbital (LUMO) gaps of the free organic ligand and the MFM‐300(M) frameworks after coordination with the Al(III), Sc(III) and In(III) centers. The values were determined using the direct Tauc method,^[^ ^57^ ^]^ remain very similar, ranging between 3.71 and 3.83 eV (see Section S2.3). This result emphasizes the idea that the ligand orbitals dominate the frontier orbitals of the MOF indicating that the emission remains centered on the ligand.^[^ ^57^ ^]^ It is worth to point out, in the case of MFM‐300(Cr), it was not possible to determine with confidence the HOMO‐LUMO energy based on the available experimental data, due to the absence of a well‐defined absorption edge, which prevents the application of the Tauc method.

On the other hand, electronic configurations of each trivalent cation can also provide insights for our hypotheses on the large difference in fluorescence emission intensities observed in the MFM‐300(M) series. As Al(III) lacks electrons in the d orbitals, possible d‐d transitions, which are typically non‐radiative, and contribute to energy dissipation, are not possible. casein contrast, Cr(III), with a partially filled 3d^3^ configuration, can undergo such d‐d transitions, facilitating non‐radiative decay pathways, which contributes to the significantly lower emission intensity observed in MFM‐300(Cr) compared to the other materials in the series. Thus, we can conclude that the electronic configuration of the metal combined with the charge density of the metal centers are strongly related to the modulation of the fluorescence in this type of materials.

### 2.3 Density Functional Theory (DFT) Calculations

**Figure 4:**
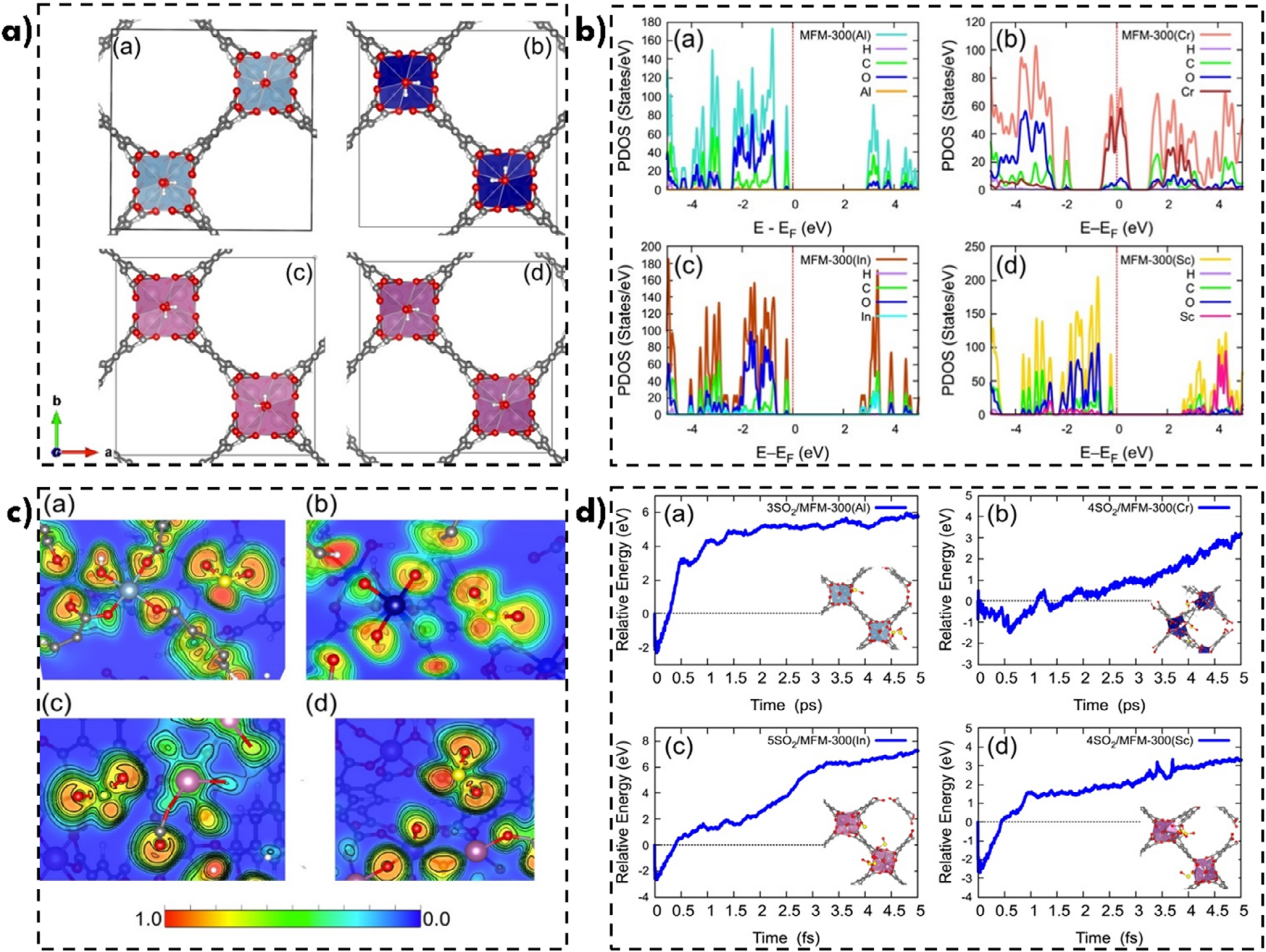
a) Relaxed atomic geometries for: MFM‐300(Al), MFM‐300(Cr), MFM‐300(In), and MFM‐300(Sc) structures. Colour code: C (grey), O (red), Al (blue), Cr (Navy), In (pink), and Sc (violet). b) Partial density of electronic states (PDOS) for: MFM‐300(Al), MFM‐300(Cr), MFM‐300(In), and MFM‐300(Sc) structures. c) Electronic local function (ELF) in at plane M‐SO_2_ for: MFM‐300(Al), MFM‐300(Cr), MFM‐300(In), and MFM‐300(Sc) structures. d) Molecular dynamics profile for: 3SO_2_/MFM‐300(Al), 4SO_2_/MFM‐300(Cr), 5SO_2_/MFM‐300(In), and 4SO_2_/MFM‐300(Sc).

To verify the structural stability and electronic configuration of the MFM‐300(M) series under investigation, DFT‐based calculations were performed. The computational methodology is detailed in Section S2.4 of the SI. Periodic boundary condition DFT calculations were used to optimize the structures of MFM‐300(M = Al(III), Cr(III), In(III) and Sc(III)). Due to the significant impact of M^3+^ cation insertion on the unit cell volume, full cell relaxation was carried out. The relaxed parameters and structures are presented in **Figure** **4**a and Table S3, Supporting Information, respectively. In all cases, the metal centers maintain the structural rigidity of the MOF while preserving open regions suitable for interactions with target gas molecules (SO_2_ in this study). Conversely, MFM‐300(In) and MFM‐300(Sc) based systems exhibit larger pore volumes and sizes compared to their MFM‐300(Al) and Cr based counterparts. These trends in behavior is analogous to the experimental observations presented in Table 1.

Computationally determined characteristic bond distances of the MFM‐300(M) materials are summarized in Table S4, Supporting Information. Notably the M(III)─O bond length parameter is larger with respect to the M(III)‐OH interaction, except for MFM‐300(Cr). Compared to the experimental bond distances (see Figure 3), the most pronounced bond contractions determined computationally are observed for the Al(III) and Cr(III) centers (see Table S4, Supporting Information). The electronic properties of these structures were examined through calculations of the projected density of states (PDOS), as showed in Figure 4b. According to PDOS calculations the MFM‐300(M) series (M = Al(III), In(III), Sc(III)) present an energy gap greater than 2.5 eV. Notably MFM‐300(Al) exhibits the largest band gap (>3.0 eV), which progressively decreases in the In(III) and Sc(III) systems. However, MFM‐300(Cr) exhibits semi‐metallic or hall‐metal behavior. These calculations corroborate the electronic transition hypothesis. No energy gap is observed for MFM‐300(Cr) according to DFT+D3 calculations. It is evident that virtual electronic states emerge above the Fermi level especially in the conduction bands for In(III) and Sc(III). The absence of a band gap for MFM‐300(Cr) may be strongly related to its low emission (Figure 2). It is worth noting that the contributions from O and C atoms predominate significantly near the Fermi level. This is the exception for the MFM‐300(Cr) system, where the Fermi level is half that of the Cr‐PDOS contributions. In the case of MFM‐300(Al), the absence of d electrons is evident as this material does not preserve electronic states in the energy window analyzed.

Subsequently, we evaluated the interaction of the SO_2_ molecule on the metal centers. The electronic localization function (ELF) for molecular geometry optimization is presented in Figure 4c. Results show that a covalent bond is not formed in all cases. However, the influence of the metal centers generates a slight distortion in the ELF of the SO_2_ molecule, particularly in the cases of Al(III), In(III) and Sc(III) as follows. For MFM‐300(Sc), the ELF contour shows a clear absence of electron localization over the Sc(III) ion (Figure 4c(a)). However, an electronic effect is evident, as it induces a distortion in the SO_2_ molecule; this corroborates the calculated adsorption energies which suggest an interaction with the SO_2_ molecule, that is, physisorption, within the MOF structure (see Table S5, Supporting Information). The calculations show that the strongest SO_2_‐MOF interaction occurs for MFM‐300(In), with an adsorption energy of −0.28 eV, while the weakest interaction is observed for SO_2_/MFM‐300(Sc), with an energy of −0.12 eV. Despite these values, in both cases the SO_2_ molecule is located closer to the metal center, compared to the MFM‐300(Sc and Cr) systems (4.17 and 3.91 Å, respectively). The negative adsorption energies clearly indicate that the SO_2_ adsorption process at the metal centers is exergonic in nature; this is corroborated by the experimental results.

Furthermore, the optical properties of the MFM‐300(M) MOFs were evaluated through calculations both before and after SO_2_ adsorption via Time Dependent (TD)‐DFT calculations. Figure S10, Supporting Information depicts the theoretical absorption spectra, clearly showing the response of the different metal‐containing centers in the blue region of the spectrum. This behavior correlates with the emission energies shown in Figure 2. It is worth pointing out that the spectra remain unchanged after SO_2_ adsorption, indicating that the optical properties of the systems are preserved before and after the adsorption process. In the specific case of the MFM‐300(Cr) system, a distinct behavior is observed, which may be attributed to its electronic structure, characterized as semi‐metallic.

Based on the high capacity of MFM‐300(M) materials to accommodate SO_2_ molecules, their structural stability was assessed through molecular dynamics (MD) simulations. The SO_2_ molecule retention capacity was obtained from the data in Table 1. Figure 4d depicts the molecular dynamics trajectories, simulated over a duration of 5 ps with a time step of 2.5 fs at T = 300 K. Consistent with their SO_2_ retention capacity, all MFM‐300(M) MOFs evolve to maintain the SO_2_ molecules in close proximity to the metal centers. According to the relative energy profiles (Figure 4d), the energy intervals exceed 3 eV, a value considered sufficient to permit desorption of the SO_2_ molecules. Furthermore, the structural stability of the MOFs after reaching 5 ps of the simulation is observable. Interestingly, in the MD simulations, the SO_2_ molecules do not exhibit significant intermolecular chemical interactions, as their interactions are primarily dominated by coordination with the metal centers. In the specific case of the 5SO_2_/MFM‐300(In) system, SO_2_‐SO_2_ interactions are observed during the molecular dynamics simulations due to their spatial proximity. However, these interactions are not sufficiently attractive to induce desorption from the surface of the MOF. Therefore, both the structural stability and the interactions at the metal centers support the presence of SO_2_ molecules, as reported in Table 1. To provide a clearer visualization of these results, molecular dynamics simulation videos are included in the supplementary multimedia material. It is worth mentioning that the 3SO_2_/MFM‐300(Al) system retains only one SO_2_ molecule by the end of the molecular dynamics simulation.

Remarkably, the computational results are consistent with the experimental observations, for example, the SO_2_ uptake capacities obtained via MD simulations correlate with the reported experimental uptake values (Table 1). Moreover, the preservation of the framework integrity throughout the MD trajectories and the structural rigidity observed in the DFT‐optimized models are consistent with the absence of structural collapse observed in the ex situ PXRD patterns after SO_2_ exposure (Figure S2, Supporting Information).

### 2.4 SO_2_ Detection Measurements

**Figure 5:**
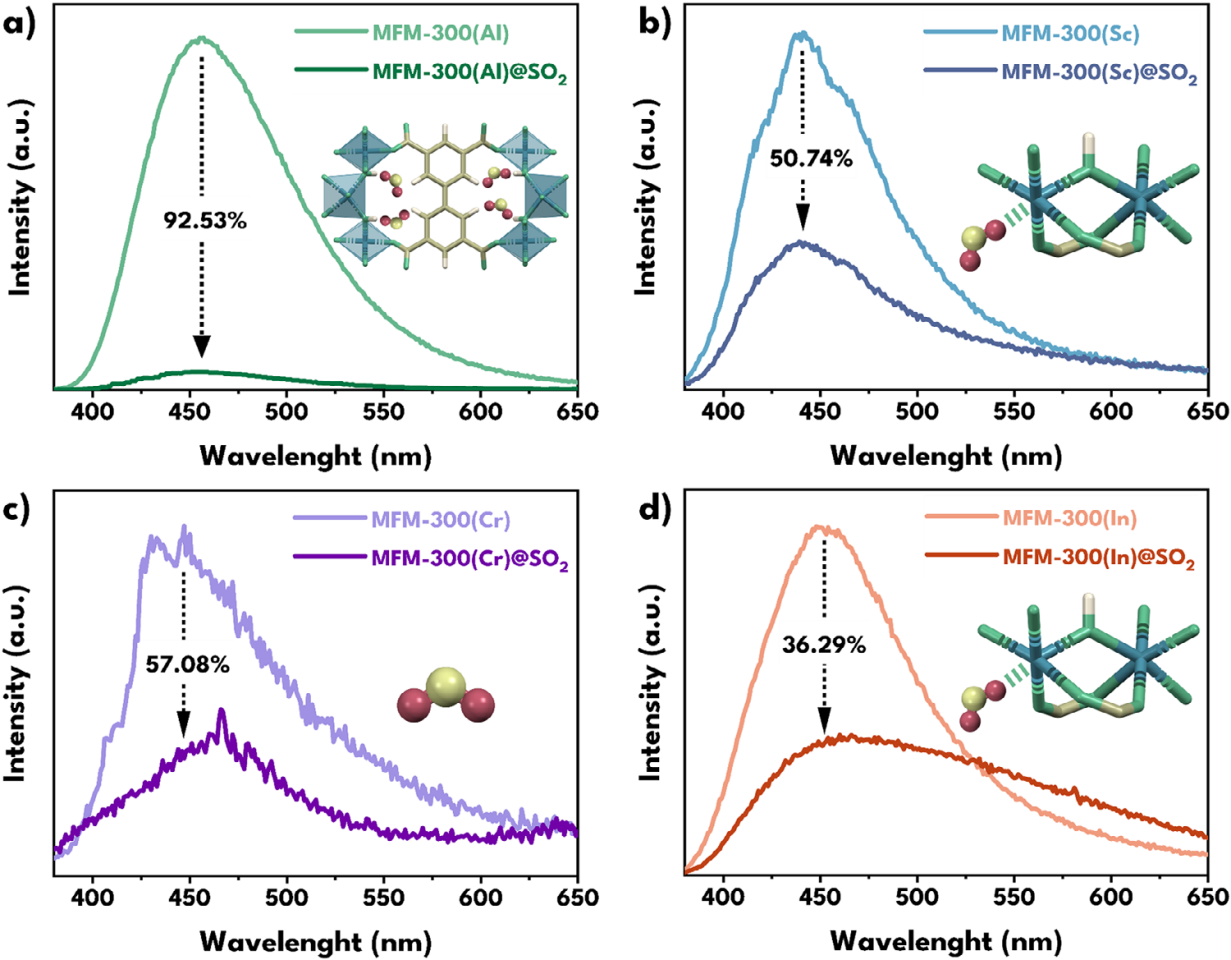
Comparison of fluorescence quenching (at λ_ex_ = 360 nm) after SO_2_ saturation of a) MFM‐300(Al) (green), b) MFM‐300(Sc) (blue), c) MFM‐300(Cr) (purple), and d) MFM‐300(In) (orange).

The interesting fluorescence characteristics of MFM‐300(M), the performance of these materials as SO_2_ adsorbents, as well as the findings of calculations and simulations, led us to investigate the effect of SO_2_ on the fluorescence of these materials (**Figure** **5**). The fluorescence spectra of each activated material and after saturation with SO_2_ were measured, showing in all cases that the SO_2_‐framework interaction leads to a decrease in fluorescence intensity, corroborated by the quantum yields QY of the saturated materials (**Table** **2**), which exhibit reductions ranging from ≈99% (for MFM‐300(Al), from 44.89% to 0.36%) to ≈57% (for MFM‐300(Cr), from 0.49% to 0.21%). Note that MFM‐300(Al) is the best performer, as it exhibits the highest quantum yield in the activated state (44.89%) and also the strongest fluorescence quenching upon SO_2_ exposure, with a 99.2% decrease down to 0.36%).

**Table 2:** Quantum Yield of MFM‐300(M) series.

Material	Activated [%]	SO_2_‐saturated [%]
H_4_BPTC	0.31	—
MFM‐300(Al)	44.89	0.36
MFM‐300(Sc)	1.07	0.53
MFM‐300(Cr)	0.49	0.21
MFM‐300(In)	2.38	1.48

The difference in the SO_2_‐induced response is a consequence of the interactions with the ligand and the reversible rearrangement between the metal centers and the coordinating carboxylate groups. MFM‐300(In) and MFM‐300(Sc) have been previously reported to exhibit hemilabile bonds between the metal centers and the carboxylates. For example, in MFM‐300(Sc), Sc‐N interactions occur upon exposure to NH_3_,^[^ ^31^ ^]^ Sc‐S for hydrogen sulfide (H_2_S),^[^ ^58^ ^]^ and Sc‐CN for HCN.^[^ ^33^ ^]^ These mechanisms were corroborated by DFT calculations showing charge redistribution and elongation of the Sc─O bond during gas adsorption;^[^ ^33^ ^]^ and, for MFM‐300(In), In‐CN interactions occur with HCN,^[^ ^59^ ^]^ and In‐S due to the exposition with H_2_S.^[^ ^58^ ^]^

In contrast, we hypothesize herein that the stronger Al─O bonds in MFM‐300(Al) inhibit the formation of transient open metal sites, even with the inclusion of SO_2_ guest molecules. This is corroborated by ELF, which allowed us to visualize how the presence of SO_2_ affects the electronic environment in each of the MFM‐300(M) materials. In MFM‐300(Al), electronic distortion occurs in the SO_2_ molecule without showing an electronic localization on the metal center, indicating that the interaction occurs mainly with the functional groups of the ligands, such as the *µ*_2_‐OH groups and the aromatic rings of BPTC^4−^. In addition, the Al(III) analogue shows the highest M‐SO_2_ distance (Table S5, Supporting Information). In contrast, in MFM‐300(In) and MFM‐300(Sc), where the metal‐ligand bonds are more hemilabile, the ELF suggests a greater proximity and influence of the metal center on the SO_2_, which points to the formation of partially open sites, showing shorter M‐SO_2_ distances, in these cases, we hypothesize that reversible Sc(III)···O═S═O and In(III)···O═S═O interactions occur, where SO_2_ interacts with the semi‐open metal site through its O‐atom, similarly to other MOFs with open metal sites such as MFM‐170 and UiO‐66‐Cu^II^.^[^ ^60^, ^61^ ^]^ Finally, in the case of MFM‐300(Cr), the ELF shows a profile with no significant interaction between SO_2_ and the metal nor the ligand, which, together with its semi‐metallic behavior evidenced in the PDOS, explains the low basal fluorescence, probably due to the rapid dissipation of the excited energy via internal non‐radiative transitions.

Thus, in MFM‐300(Al), the interaction with SO_2_ relies on static supramolecular interactions, such as the formation of hydrogen bonds between the ─OH groups of the framework and SO_2_, as well as C─H···O interactions with the aromatic rings of the ligand, as reported by Yang et al.^[^ ^25^ ^]^ This type of direct interaction with the functional groups of the organic ligands in the network could result in a local redistribution of the electron density that can favor the non‐radiative deactivation of the excited state of the ligand, which means that the fluorescence quenching is more remarkable in MFM‐300(Al) in comparison with its analogues. In other words, the response of MFM‐300(Al) to SO_2_ adsorption is more influenced by such specific and rigid interactions, rather than depending on the hemilability of the metal‐ligand bond that characterizes other MFM‐300 series analogues.

### 2.5 Quenching Mechanisms in MFM‐300(M)

SO_2_ exhibits a significant dipole moment, being a polar molecule with a lone pair of electrons on the oxygen atoms and a sulphur atom with a partially positive character. This makes it susceptible to electrostatic interactions and charge transfer in the presence of polar centers or Lewis acids. On the other hand, the high charge density of Al(III) and the polarization of the ─OH groups of MFM‐300(Al) facilitate the acceptance of electron density from host molecules such as SO_2_. Thus, the O‐atom (partially negative, δ‐) of SO_2_, interacts with the H‐atom of the *µ_2_*‐OH ligand of the framework via hydrogen bonds. In this interaction SO_2_ acts as an electron donor to the framework through strong dipolar coupling.^25^ In addition to that, the O‐atoms of SO_2_, also form weak C─H···O type interactions with the aromatic rings of the ligand, allowing additional coupling between the gas and the π‐conjugated system of the ligand, facilitating electron density delocalization. Electron density transfer from the lone pairs of the SO_2_ oxygens to the π‐system of the ligand, or alternatively to the empty orbitals of the Al(III) atoms, and electronic redistribution in the MFM‐300(Al) framework, could be deactivating certain excited states responsible for the fluorescence, causing the experimentally observed fluorescent quenching.

An FTIR spectrum was performed on MFM‐300(Al) saturated with SO_2_ (Figure S5, Supporting Information), showing significant changes between the host and host‐guest systems which supports the interaction between SO_2_ and the ligand. First, two small bands (absent in MFM‐300(Al)) appear at 1339 and 1011 cm^−1^ in the SO2 saturated material demonstrating the asymmetric and symmetric stretching of the S═O bond.^[^ ^62^, ^63^ ^]^ These bands confirm that SO_2_ is adsorbed within the pores of MFM‐300(Al) (Figure S5, Supporting Information). Additionally, the peak at 3692 cm^−1^, corresponding to the *µ*_2_‐OH ligand,^[^ ^32^ ^]^ decreases in intensity and broadens in the presence of SO_2_, suggesting a relatively strong interaction between SO_2_ and the Al‐O(H)‐Al moiety (Figure S6, Supporting Information).^[^ ^35^ ^]^ Additionally, the asymmetric stretching vibration of the carboxylate shows a visible shift from 1586 to 1580 cm^−1^ along with a change in intensity (Figure S5, Supporting Information).

To further understand how SO_2_ adsorption perturbs the electronic environment of the frameworks, ultraviolet‐visible (UV–vis) spectroscopy and band gap analysis were performed on the saturated materials. Slight decreases in the absorbances can be observed in all cases (Figure S8, Supporting Information), suggesting guest‐induced polarization of the ligand environment. In MFM‐300(Al) and MFM‐300(Cr), the small decrease in absorption intensity is presumably due to polar interactions between SO_2_ and functional groups such as *µ*_2_‐OH, carbonyls and aromatic rings, these interactions can locally polarize the conjugated system and reduce the number of optically active sites, reducing slightly the absorption efficiency without altering the nature of the frontier orbitals. In contrast, MFM‐300(Sc) and MFM‐300(In) show more pronounced decreases, which are attributed to the presence of semi‐open metal sites that allow the SO_2_ to approach the metal centers and can induce weak charge transfer and polarization effects, leading to a partial redistribution of the electron density in the ligand and a more significant reduction of the π→π* transition intensity. When determining the HOMO‐LUMO energy gap using the direct Tauc method (Figure S9, Supporting Information),^[^ ^64^ ^]^ minimal variations in the gaps are observed after SO_2_ adsorption, ranging from 0.01 to 0.09 eV (Table S2, Supporting Information). This indicates that the incursion of SO_2_ into the framework does not drastically alter the position of the frontier orbitals and that the charge transfer between the host and the framework occurs in a localized way. The small modifications can be attributed to a slight redistribution of the electron density upon SO_2_ adsorption, without changing the emission mechanism, which remains centered on the ligand.

**Figure 6:**
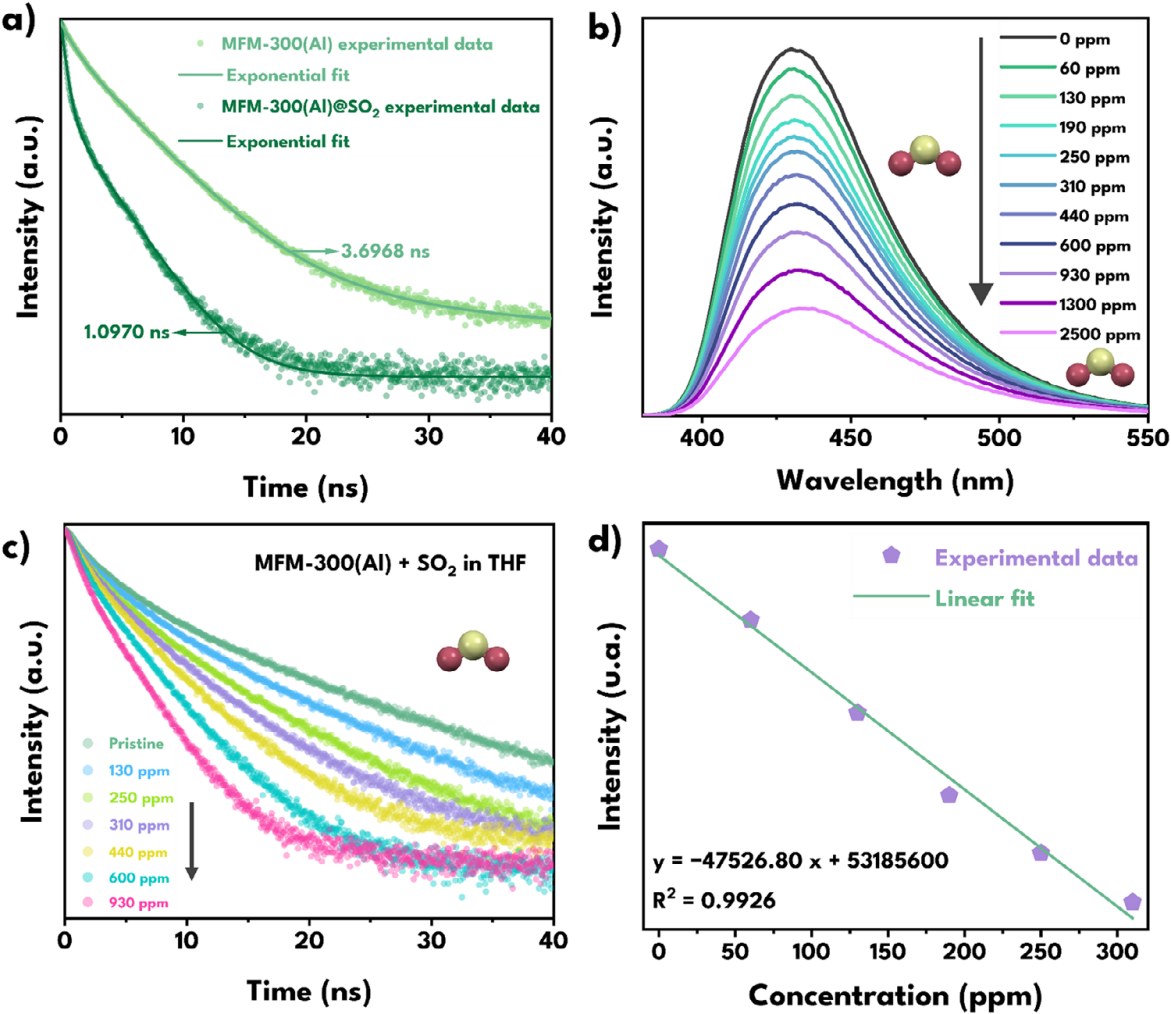
a) Time‐resolved photoluminescence decay profiles at λ_em_= 455 nm of solid‐state MFM‐300(Al) before and after SO_2_ exposure. b) Fluorescence response of MFM‐300(Al) toward SO_2_ in a THF suspension. c) Time‐resolved photoluminescence decay profiles at λ_em_= 455 nm of dispersed MFM‐300(Al) in SO_2_ solutions in THF. d) Calibration curve used for the determination of the LOD.

TRPL experiments on MFM‐300(Al), the analogue with the highest fluorescence quantum yield and the most significant quenching upon SO_2_ exposure, showed that, after exposure to SO_2_, there was a notable decrease in the fluorescence lifetime ⟨τ⟩ from 3.79 to 1.08 ns (**Figure** **6**a), supporting the hypothesis that non‐radiative decay pathways are promoted by host‐guest interactions in this system. The multi‐exponential analysis of the decay spectra (Table S6, Supporting Information) showed that, in the absence of SO_2_, τ_1_ ≈ 1.00 ns contribute only a_1_ ≈14%, while the intermediate and long contributions (τ_2_ ≈ 3.40 ns, a_2_ ≈ 59% and τ_3_ ≈ 6.20 ns, a_3_ ≈ 26%) dominate the emission. However, after SO_2_ adsorption, the fast component τ_1_ ≈ 0.09 ns increase dramatically to a_1_ ≈ 52%, while τ_2_ and τ_3_ decrease in contribution and intensity (τ_2_ ≈ 1.15 ns, a_2_ ≈ 24% and τ_3_ ≈ 3.27 ns, a_3_ ≈ 23%). This change reflects a strong redistribution toward ultrafast processes derived from non‐radiative mechanisms.^[^ ^65^ ^]^ After saturation with SO_2_, the DAS (Figure S21b, Supporting Information) shows a clear redistribution of τ_1_, which increases and also dominates the spectrum in practically the entire 420‐600 nm range, especially in the green‐red zone; at the same time, τ_2_ and τ_3_ remain close, retaining weak amplitudes.

While static quenching is usually characterized by a decrease in emission intensity without affecting the fluorescence lifetime,^[^ ^66^ ^]^ the time‐resolved photoluminescence data for MFM‐300(Al) reveal a strong decrease in lifetime values upon SO_2_ adsorption. This indicates that the emission is not merely suppressed via ground‐state complexation, but rather that a dynamic non‐radiative deactivation mechanism is induced upon SO_2_ interactions.^[^ ^67^ ^]^ This is confirmed by the Stern‐Volmer plot (Figure S12, Supporting Information), in which MFM‐300(Al) shows highly linear behavior, with a coefficient of determination R^2^ of 0.9952. This result indicates that only one type of quenching mechanism is operating, furthermore, this linearity suggests that the turn‐off process is predominantly dynamic.^[^ ^68^, ^69^ ^]^

The unchanged bandgap (from 3.83 to 3.82 eV, Figure S9, Supporting Information) and minimal UV–vis spectral changes (Figure S8, Supporting Information) suggest that the photoexcited state remains centered on the ligand but is efficiently quenched via local interactions through strong hydrogen bonding between SO_2_ and *µ*_2_‐OH and the aromatic groups in the framework. These specific host‐guest interactions with SO_2_ interfere with the excited state of the ligand, promoting an ultrafast non‐radiative decay pathway that becomes the main deactivation route after SO_2_ adsorption.

For MFM‐300(Sc) and MFM‐300(In), the quenching mechanisms differ due to their known semi‐open metal site character. Both structures allow the SO_2_ molecule to approach the metal center more closely (Sc‐SO_2_ = 3.48 Å; In‐SO_2_ = 3.39 Å). In these cases, we propose a charge transfer process,^[^ ^70^ ^]^ in which electron density from the lone pairs of the O‐atom of the SO_2_ is partially transferred to the *d*‐orbitals of Sc(III) or In(III) in the excited state. These results demonstrate an effective dynamic quenching process, where the excited ligand no longer returns to the ground state via fluorescence but instead, undergoes by a rapid non‐radiative deactivation. While the HOMO‐LUMO gaps remain practically unchanged after SO_2_ adsorption, the slight decreases observed (Sc: 3.71 to 3.67 eV; In: 3.80 to 3.71 eV) may reflect local electronic redistribution at the metal sites, supported by the short M─SO_2_ distances and adsorption energies.

In contrast, MFM‐300(Cr) displays a modest fluorescence, which can be attributed to its intrinsic non‐emissive character. The Cr(III) center (3d^3^) enables spin‐forbidden d‐d transitions that promote non‐radiative decay even before SO_2_ inclusion.^[^ ^71^ ^]^ Although the ELF and DFT data show that SO_2_ does interact with the framework (Cr‐SO_2_ = 3.91 Å, E_ads_ = −0.27 eV), no significant redistribution of the frontier orbitals is observed (bandgap slightly increases from 3.67 to 3.70 eV). Therefore, the quenching observed in this case likely results from a modulation of existing non‐radiative pathways, rather than the creation of new electronic channels. SO_2_ may act as a weak perturbing species that enhances internal conversion or vibrational relaxation without directly participating in excited‐state electron transfer.

TRPL experiments for MFM‐300(Sc) (Figure S17 and Table S6, Supporting Information) showed that ⟨τ⟩ decreases from 2.73 to 1.69 ns, with an increase in the contribution of τ_1_ from 37% to 46% (τ_1_ goes from 0.46 to 0.36 ns), while τ_2_ and τ_3_ decrease in time and contribution (τ_2_: from 1.94 to 1.55 ns; τ_3_: from 7.71 to 5.59 ns). In MFM‐300(In) (Figure S19, Supporting Information) exhibits a comparable behavior, ⟨τ⟩ goes from 2.45 to 1.29 ns, increasing the contribution of the fast component τ_1_ increases from 44% to 66% (τ_1_ goes from 0.42 to 0.19 ns), while τ_2_ and τ_3_ lose contribution and are shortened (τ_2_: from 1.85 to 1.49 ns; τ_3_: from 7.63 to 6.04 ns). This indicates that, although the effect is not as extreme as in Al, the presence of SO_2_ also facilitates an increase in fast deactivation channels to the detriment of slower radiative processes. In MFM‐300(Cr) (Figure S18, Supporting Information) ⟨τ⟩ is subtly reduced from 1.42 to 1.07 ns, the τ_1_ component, which is already dominant (56%), increases even more to 73% after adsorption, and τ_2_ and τ_3_ decrease slightly in time and contribution. Since this material has an inherently low emission efficiency, exposure to SO_2_ barely increases the proportion of non‐radiative processes that were already predominant. The DAS of MFM‐300(Sc), MFM‐300(In) and MFM‐300(Cr) after SO_2_ adsorption (Figures S22–S24, Supporting Information) show that τ_1_ remains the dominant contribution throughout the entire spectrum, especially in the green‐blue region (≈540–580 nm), reflecting the increase in non‐radiative pathways.

Due to the good performance of MFM‐300(Al) in fluorescence quenching after exposure to SO_2_, experiments were carried out in suspension in THF to quantify its limit of detection (LOD) against different concentrations (Figure 6b). With this, an LOD of 51 ppm was determined (Figure 6d),^[^ ^68^ ^]^ which underlines the high sensitivity of this material toward such gas. Furthermore, TRPL experiments were performed on MFM‐300(Al) dispersed in THF after exposure to different concentrations of SO_2_ (Figure 6c). These measurements clearly show not only a drastic reduction in the average fluorescence lifetime ⟨τ⟩, but also a progressive redistribution in the durations and relative contributions of the different decay components (Table S7, Supporting Information). The pristine sample has three components, as in the solid state, with the long‐lived component dominating (τ_3_ ≈ 11.5 ns, a_3_ ≈ 66%). However, as the SO_2_ concentration increases, this long component is systematically suppressed, while the intermediate and fast components acquire a greater relative contribution. For example, at 930 ppm SO_2_, the contribution of τ_3_ decreases to ≈2%. This evolution in the fluorescence time components once again highlights the role of SO_2_ in generating non‐radiative deactivation pathways that cause fluorescence quenching.

**Figure 7:**
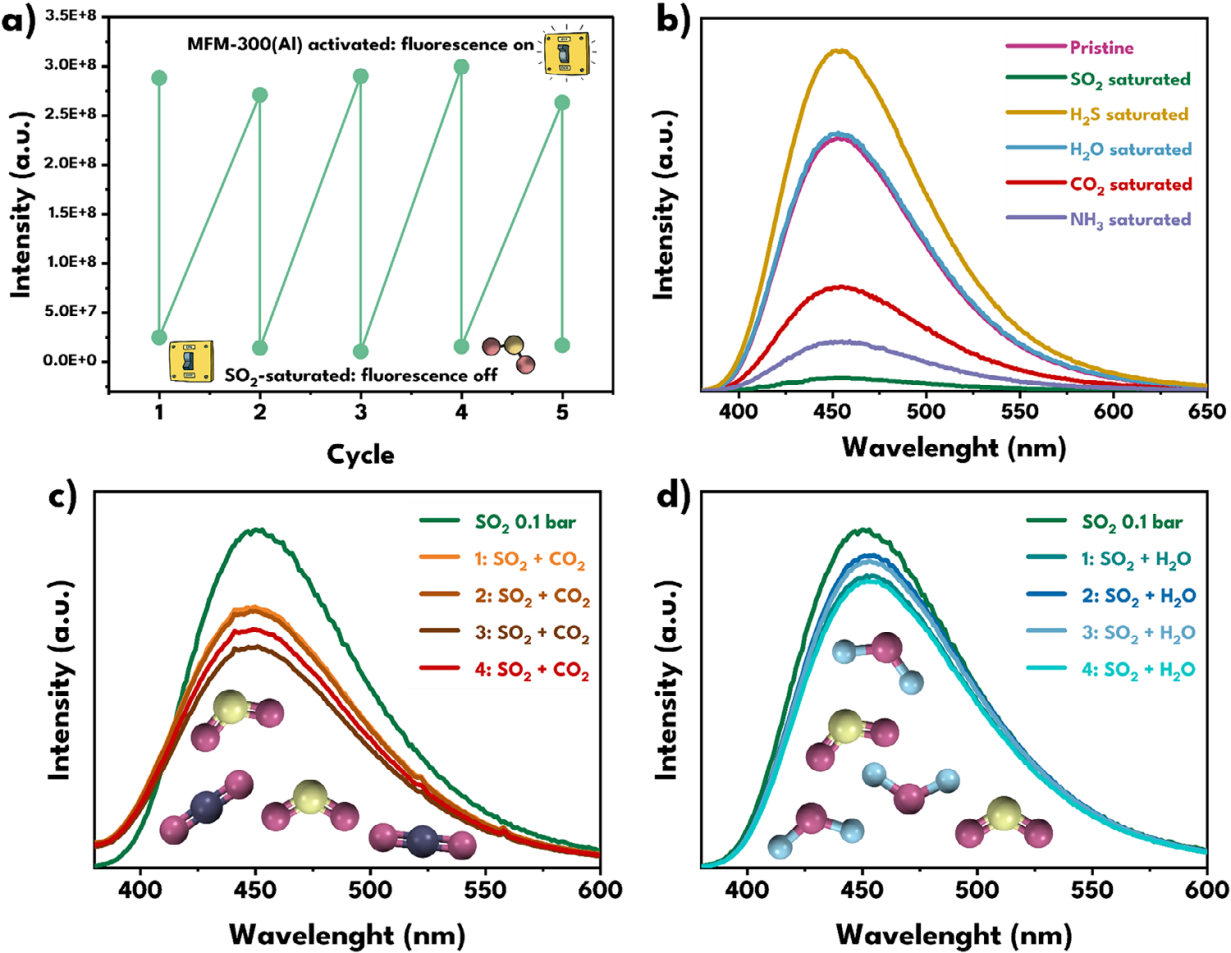
Evaluation of the fluorescent regenerability and selectivity of MFM‐300(Al) against SO_2_ in solid‐state. a) SO_2_ activation‐saturation cycles. b) Comparison of the emission spectra of the pristine material and after exposure to gases/vapor: SO_2_ (green), H_2_S (yellow), H_2_O (blue), CO_2_ (red) and NH_3_ (purple). c) Comparison of the fluorescent response to the SO_2_ + CO_2_ mixture. d) Comparison of the fluorescent response to the SO_2_ + H_2_O mixture.

The regenerability of MFM‐300(Al) in solid state was evaluated by five consecutive cycles of thermal activation (150 °C under vacuum) and saturation with SO_2_. In all cases, it was observed that the fluorescence of the pristine material was regenerated after activation, while the SO_2_‐induced quenching remained unchanged in each cycle (**Figure** **7**a). This behavior demonstrates the ability of the material to recover its photoluminescent response after the regeneration process and maintain repeatable detection performance. Additionally, the regenerated sample was characterized by PXRD, confirming that the crystalline structure of the framework remained invariant during the cycles (Figure S3, Supporting Information).

Additionally, H_2_S, NH_3_, carbon dioxide (CO_2_) and H_2_O were evaluated to explore the possible selectivity of MFM‐300(Al) for SO_2_, observing considerable quenching for CO_2_, H_2_S, and NH_3_, but lower than in the case of SO_2_. (Figure 7b). These differences can be explained by considering the polarity, electron donation/acceptance capacity, kinetic diameter, and molecular geometry of each gas molecule, as well as the type of interaction they establish with the framework.

Of all the gases, SO_2_ has higher polarity (≈1.6 D) and high polarizability, which allows it to simultaneously couple to the *µ_2_*‐OH via hydrogen bonds and to the aromatic rings of the ligand via C─H···O interactions, facilitating direct coupling with the π system and favoring highly efficient non‐radiative pathways. In addition, its kinetic diameter of ≈4.0–4.2 Å and angular geometry (O─S─O ≈ 119°) allow it to orient one oxygen toward the *µ_2_*‐OH and the other toward the organic moiety, maximizing possible interactions. On the other hand, NH_3_ has comparable polarity (≈1.5 D) but much lower polarizability; being a strong Lewis base and a strong σ donor, it interacts with MFM‐300(Al) through hydrogen bonds via its N‐atom with the *µ_2_*‐OH (*µ_2_*‐OH···N). Its kinetic diameter of 2.6 Å is smaller than SO_2_, which, together with its pyramidal geometry (H─N─H ≈ 107°), allows it to form cooperative NH_3_···NH_3_ chains within the channel, which mainly promotes cooperative aggregation rather than direct interaction with the chromophore, resulting in intense quenching but less than that of SO_2_. The H_2_S, unlike the other gases evaluated, does not induce fluorescence quenching, but rather generates an increase in emission intensity. Although this gas has low polarity (≈0.97 D) and intermediate polarizability, the explanation for this behavior does not lie solely in hydrogen bonds with *µ_2_*‐OH, but rather in chemical changes that occur after exposure of the framework to H_2_S: these changes appear to induce structural rigidification in MFM‐300(Al), which limits non‐radiative deactivation pathways, resulting in improved emissive efficiency of the material. In contrast, CO_2_, with linear geometry (O─C─O = 180°) and zero dipole moment, has a kinetic diameter of 3.3 Å, which allows it to interact with MFM‐300(Al) by accepting a hydrogen bond from *µ_2_*‐OH (*µ_2_*‐OH···O═C═O), complemented by quadrupole and dispersion interactions with the aromatic walls; however, its coupling with the chromophore is moderate due to the absence of a dipole, which limits the direct modulation of the π system, resulting in much more modest quenching. Finally, H_2_O has very low polarizability and a small kinetic diameter of 2.65 Å, which means that direct interactions with the ligand's π system are minimal, limiting its ability to modulate the emitting electronic states and this explains why the change in fluorescence is negligible compared to the other gases studied. Consequently, the higher affinity of MFM‐300(Al) for SO_2_ supports its potential as a selective and sensitive fluorescent sensor.

Once the apparent selectivity of MFM‐300(Al) for SO_2_ had been observed, an attempt was made to test the material in a practical sensing scenario, where traces of SO_2_ coexist with quantities of CO_2_ and H_2_O vapor, so the interference of these two molecules was studied. First, the fluorescence response of the material in the solid‐state exposed to 0.1 bar of SO_2_ was evaluated (Figure S14, Supporting Information), observing an 87% decrease in fluorescence (the saturated sample decreased by 92%), demonstrating the good sensitivity of the system. Subsequently, a previously activated sample of MFM‐300(Al) was exposed to a mixture of ≈0.1 bar of SO_2_ and ≈0.9 bar of CO_2_, observing that the spectrum retains its shape and maximum emission length (≈450 nm) and that a slightly lower quenching is obtained than the one observed in the 0.1 bar sample (Figure 7c), indicating that CO_2_ does not effectively compete for the sites that generate the fluorescence deactivation pathways, consistent with the single gas data that already showed much lower quenching with CO_2_ than with SO_2_. The same sample exposed to the binary gas mixture was reactivated and re‐exposed three more times to the SO_2_+CO_2_ mixture, with consistency in the response obtained, corroborating the robustness of the system. In addition, it was confirmed that the crystalline structure is maintained by PXRD (Figure S3, Supporting Information).

The same procedure was used to evaluate H_2_O interference. An activated sample was exposed to ≈0.1 bar of SO_2_ in the presence of water vapor. In this case, co‐dosing also did not reverse the quenching by SO_2_, and the response remained comparable to that of the material exposed to 0.1 bar of anhydrous SO_2_ (Figure 7d), with no shift in the maximum or appreciable broadening. The experiment was repeated three more times, demonstrating the regenerability of the material in the presence of the SO_2_+H_2_O mixture, corroborated by PXRD (Figure S3, Supporting Information). These results indicate that the photoluminescent response is dominated by SO_2_‐framework interactions and corroborate the robustness of the material under more realistic conditions.

## 3 Conclusions

This work shows a comparative analysis of the performance of the MFM‐300(M) series (M = Al(III), Sc(III), Cr(III), and In(III)) as fluorescent detectors for SO_2_, highlighting the influence of the metal center on the emission intensity and the quenching mechanism. MFM‐300(Al) proves to be the most efficient system, with significantly higher fluorescence intensity due to the lower structural dynamics in the framework resulting from the highly stable and short Al(III)─O bond, which suppresses non‐radiative deactivation pathways in the basal state, allowing clear fluorescence emission (QY: 44.89%). Upon exposure to SO_2_ a sharp fluorescence suppression occurs, attributed to specific interactions between SO_2_ and the *µ_2_*‐OH groups and aromatic rings of the ligand. Estimations of the HOMO‐LUMO gap by the direct Tauc method, obtained from UV–vis spectra, show that such quenching occurs via local interactions that favor non‐radiative processes, without requiring a global redistribution of the bandgap. In contrast, in the Sc(III) and In(III) analogues, which have proven to be highly dynamic, exhibited quenching processes mediated by charge transfer between the O‐atom of SO_2_ and the semi‐open metal sites, directly related to the hemilability of the metal‐ligand bonds in these materials. While MFM‐300(Cr) showed intrinsically low emission due to spin‐forbidden d‐d transitions.

DFT electronic structure calculations and molecular dynamics simulations support these findings, showing that the SO_2_‐MOF interaction occurs mainly by physisorption in the MFM‐300(M) materials, with exergonic adsorption energies in all cases. ELF shows that in MFM‐300(Al), the interaction with SO_2_ is limited to the ligand environment, no interaction with the metal center occurs, supporting the model of quenching by localized interaction with the ligands. In contrast, in MFM‐300(Sc) and MFM‐300(In), ELF reveals enhanced electronic distortion close to the metal, consistent with charge transfer mechanisms mediated by the semi‐open metal sites.

TRPL experiments reveal a significant decrease in the emission lifetimes of MFM‐300(Al) in solid‐state and dispersed in THF after SO_2_ exposition, highlighting the growth of the fast decay component suggesting a dynamic quenching mechanism derived from the activation of highly efficient non‐radiative deactivation pathways induced by the host, also confirmed by observing the increase in the amplitude of τ_1_ in the DAS after SO_2_ adsorption. For MFM‐300(Al) a LOD of 51 ppm was determined for SO_2_ in a dispersed medium in THF. MFM‐300(Al) showed selectivity toward SO_2_ versus CO_2_, H_2_O, H_2_S, and NH_3_. Furthermore, tests under mixed gas conditions (SO_2_ + CO_2_ and SO_2_ + H_2_O) and consecutive adsorption‐desorption cycles confirmed that the response of MFM‐300 (Al) remains unchanged under more challenging conditions, confirming both the preservation of its crystalline structure and the reproducibility of its fluorescent signal, which highlights this material as a robust platform. Overall, this work not only showcases the potential of MFM‐300(Al) as a good‐performance and selective SO_2_ detector but also establishes a conceptual framework for the rational design of MOF‐based optical detectors with tunable responses through strategic control of the metal center.

## 4 Experimental Section

### Materials

SO_2_ All reagents and solvents were purchased from commercial suppliers and used without further purification. All water was deionized.

### Synthesis

The synthetic methods for MFM‐300(M) materials were the same as previously reported:

*MFM‐300(Al)*:^[^ ^34^ ^]^ Aluminium chloride hexahydrate, AlCl_3_·6H_2_O, (250 mg, 1.04 mmol) and biphenyl‐3,3′,5,5′‐tetracarboxylic acid, H_4_BPTC, (85 mg, 0.26 mmol) were dissolved in deionized water (15 mL) and transferred to a 35 mL vial. The vial was sealed and the contents were stirred to facilitate dissolution of the metal salt. The reaction mixture was then placed into a CEM Discover microwave cavity and heated to 210 °C for 10 min (300 W, maximum forward power) under autogenous pressure with stirring. After cooling in the microwave cavity with air, the resulting white suspension was centrifuged (4200 rpm, 20 min), washed with distilled water (≈50 mL), and the washing step was repeated twice. The final solid was dried in an oven at 50 °C for 18 h and allowed to rehydrate in ambient atmosphere for 8 h.

*MFM‐300(Sc)*:^[^ ^26^ ^]^ Scandium triflate, Sc(CF_3_SO_3_)_3_, (30 mg, 0.061 mmol) and H_4_BPTC (10 mg, 0.030 mmol) were mixed in THF (4.0 mL), DMF (3.0 mL), water (1.0 mL) and HCl (36.5 %, aq., 2 drops). The resultant slurry mixture was stirred. The solution was then placed in a 15 mL pressure tube (Ace Glass Inc.) and heated in an oil bath to 75 °C for 72 h. The tube was cooled down to room temperature, and the colorless crystalline product was separated by filtration, washed with DMF (5.00 mL) and dried in air.

*MFM‐300(Cr)*:^[^ ^35^ ^]^ Chromium chloride hexahydrate, CrCl_3_·6H_2_O, (283 mg, 1.06 mmol) and H_4_BPTC (70 mg, 0.212 mmol) were dissolved in water (10 mL) to which HCl (12 %, aq., 1.5 mL) was then added. The reaction mixture was transferred to a 23 mL autoclave which was sealed and heated to 210 °C for 72 h. The resulting blue powder was separated by filtration and washed repeatedly with acetone and stored in acetone until required.

*MFM‐300(In)*:^[^ ^36^ ^]^ Indium nitrate pentahydrate, In(NO_3_)_3_·5H_2_O, (585 mg, 1.50 mmol) and H_4_BPTC (330 mg, 1.00 mmol) and were mixed and dispersed in a mixture of DMF (20 mL), MeCN (10 mL) and HNO_3_ (65 %, aq., 1.0 mL) placed in a 15 mL pressure tube (Ace Glass Inc.). The resultant slurry mixture was stirred until complete dissolution occurred. The solution was then heated at 80 °C for 48 h. The resultant white precipitate was separated by filtration and then washed with DMF and dried briefly in air.

### Analytical Instruments—Powder X‐Ray Diffraction

PXRD patterns were measured on a Siemens Diffractometer model D5000, with CuKα1 radiation (l = 1.5406) using a nickel filter with a step scan of 0.02° and a scan rate of 0.08° min^−1^. Scans were conducted between 5° and 35° 2*θ* and data were plotted using Match! (Crystal Impact) software.

### Fourier‐transform Infrared Spectroscopy

IR spectra were acquired using a FT‐IR spectrometer by Thermo Fisher Scientific model Nicolet 6700 equipped with an ATR accessory. Measurements were taken at 25 °C, from 4000 to 400 cm^−1^ and data were plotted using OMNIC (Thermo Scientific) software.

### Solid‐state Ultraviolet‐Visible Spectroscopy

UV–vis absorption measurements were performed from 200 to 800 nm using a Shimadzu spectrophotometer UV‐2600 equipped with an ISR‐2600Plus integrating sphere and a BaSO_4_ blank. Data plotted using UVProbe (Shimadzu) software.

### Fluorescence Spectroscopy

Fluorescence spectra were collected on a FS5 Edinburgh Instruments Spectrofluorometer using a continuous wave 150 W ozone‐free xenon arc lamp at room temperature, coupled with the SC‐10 Solid‐state and SC‐05 Standard Cuvette sample holder. The solid‐state samples were packed into quartz sample holders and positioned into the instrument. Samples dispersed in THF were measured in quartz cuvettes. All spectra were acquired at ambient conditions (≈26 °C). Emission measurements were carried out using an excitation wavelength of 360 nm, with a LP‐395 filter on the detector side to remove any remaining light from the excitation source. Emission spectra were collected with a step size of 1 nm and a dwell time of 0.1 s. The excitation bandwidth was set at 1.00 nm, and the emission bandwidth for the detector at 1.00 nm. Spectra plotted using Fluoracle (Edinburgh Instruments) software.

### Time‐Resolved Photoluminescence Spectroscopy

TRPL spectra were measured in an Edinburgh Instruments FS5 Spectrofluorometer using a 375 nm laser, with an excitation bandwidth of 0.01 nm and an emission bandwidth of 1 nm, at an emission wavelength of 450 nm. Materials were measured before and after exposure to SO_2_. Spectra plotted using Fluoracle (Edinburgh Instruments) software.

### Custom Ex Situ SO_2_ Adsorption System

The ex situ SO_2_ adsorption system (Figure S1, Supporting Information) contains two principal parts:

- The gas generator, in which sodium sulfite (Na_2_SO_3_) is added to a two‐necked ball flask,^[^ ^1^ ^]^ one neck is capped with a rubber stopper through which concentrated sulfuric acid (H_2_SO_4_) is injected with a glass syringe,^[^ ^2^ ^]^ while the other neck is connected to the saturation chamber.
- The saturation chamber, made of a round‐bottomed flask,^[^ ^3^ ^]^ is connected to a vacuum line^[^ ^4^ ^]^ and a pressure gauge.^[^ ^5^ ^]^

To start the SO_2_ saturation process, a sample of MOF (about 10 mg) in a 1.5 mL glass vial was activated in a sand bath at 150 °C under vacuum for 12 h. The vial was then placed in the saturation chamber, and the adsorption system was evacuated with a vacuum line. Next, SO_2_ gas was generated by dripping concentrated H_2_SO_4_ (98%, aq.) over solid Na_2_SO_3_, the MOF sample was left continuously exposed to the gas for 2 h.

## Conflict of Interest

The authors declare no conflict of interest.

## Supporting information



Supporting Information

## Data Availability

The datasets supporting this article, including fluorescence spectra, PXRD patterns, FTIR data, UV–vis and TRPL measurements, as well as DFT and MD simulation outputs, are provided in the Supplementary Information (SI). Additional raw data is available from the University of Leeds repository, [hyperlink to be added once available]. The data that support the findings of this study are available from the corresponding author upon reasonable request.
