# Supplementary material for: Fluorescent Sensing of SO2 by MFM‐300(M) Metal–Organic Frameworks: Influence of Semi‐Open Metal Centres
Source: Small. 2025 Sep 1;21(42):e07448. doi: 10.1002/smll.202507448 (PMC12548010; doi:10.1002/smll.202507448)
Supplement: Supplementary file 1 — Supporting Information [file SMLL-21-e07448-s001.docx]

Supporting Information

Fluorescent sensing of SO_2_ by MFM-300(M) metal–organic frameworks: influence of semi-open metal centres

Valeria B. López-Cervantes,‡^a^ Hashim Alhashimi,‡^b^ Christian A. Celaya,‡^c^ M. Solórzano,^c^ Marco L. Martínez,^a,d^ Yoarhy A. Amador-Sánchez,^e^ Evandro Castaldelli,^b^ Edward Lester,^b^ Ricardo A. Peralta,^e^ Enrique Lima,^a^ Diego Solis-Ibarra,^a^ Sihai Yang,^f^ Ilich A. Ibarra,^a^* and Andrea Laybourn^b,g^*

a) Laboratorio de Fisicoquímica y Reactividad de Superficies (LaFReS), Instituto de Investigaciones en Materiales, Universidad Nacional Autónoma de México, Circuito Exterior s/n, CU, Coyoacán, Ciudad de México, México.

b) Advanced Materials Research Group, Faculty of Engineering, University of Nottingham, Nottingham, UK.

c) Centro de Nanociencias y Nanotecnología, Universidad Nacional Autónoma de México, Km 107 Carretera Tijuana-Ensenada, Ensenada, Baja California, Mexico.

d) ESIQIE – Instituto Politécnico Nacional, Avenida IPN UPALM Edificio 7, Zacatenco, Ciudad de México, Mexico.

e) Departamento de Química, Universidad Autónoma Metropolitana-Iztapalapa, Av. Ferrocarril San Rafael Atlixco 186, Col. Leyes de Reforma 1A Sección, Iztapalapa, 09310, Ciudad de México, México.

f) College of Chemistry and Molecular Engineering, Beijing National Laboratory for Molecular Sciences, Peking University, Beijing, China.

g) Institute of Process Research and Development & School of Chemistry, University of Leeds, Leeds, UK

**S1. Experimental details**

**S1.1. Custom *ex-situ* SO_2_ adsorption system**


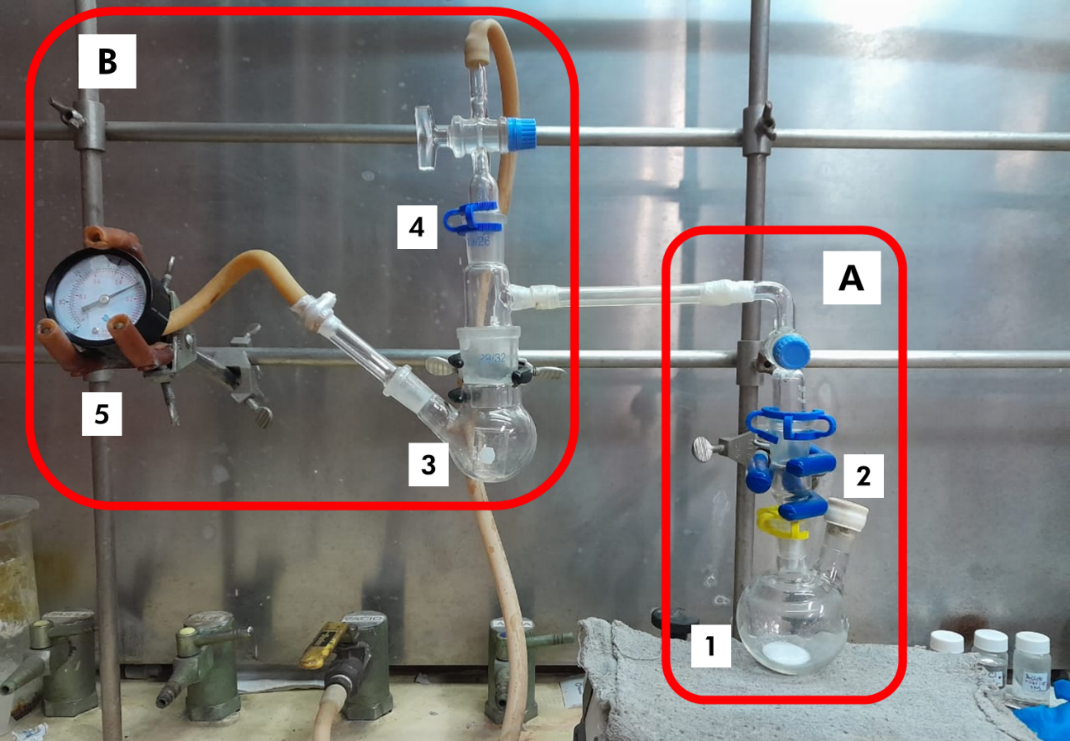


**Figure S1.** *Ex*-*situ* SO_2_ generator homemade system.

**S2. Characterization of MFM-300(M) materials before and after SO_2_**

**
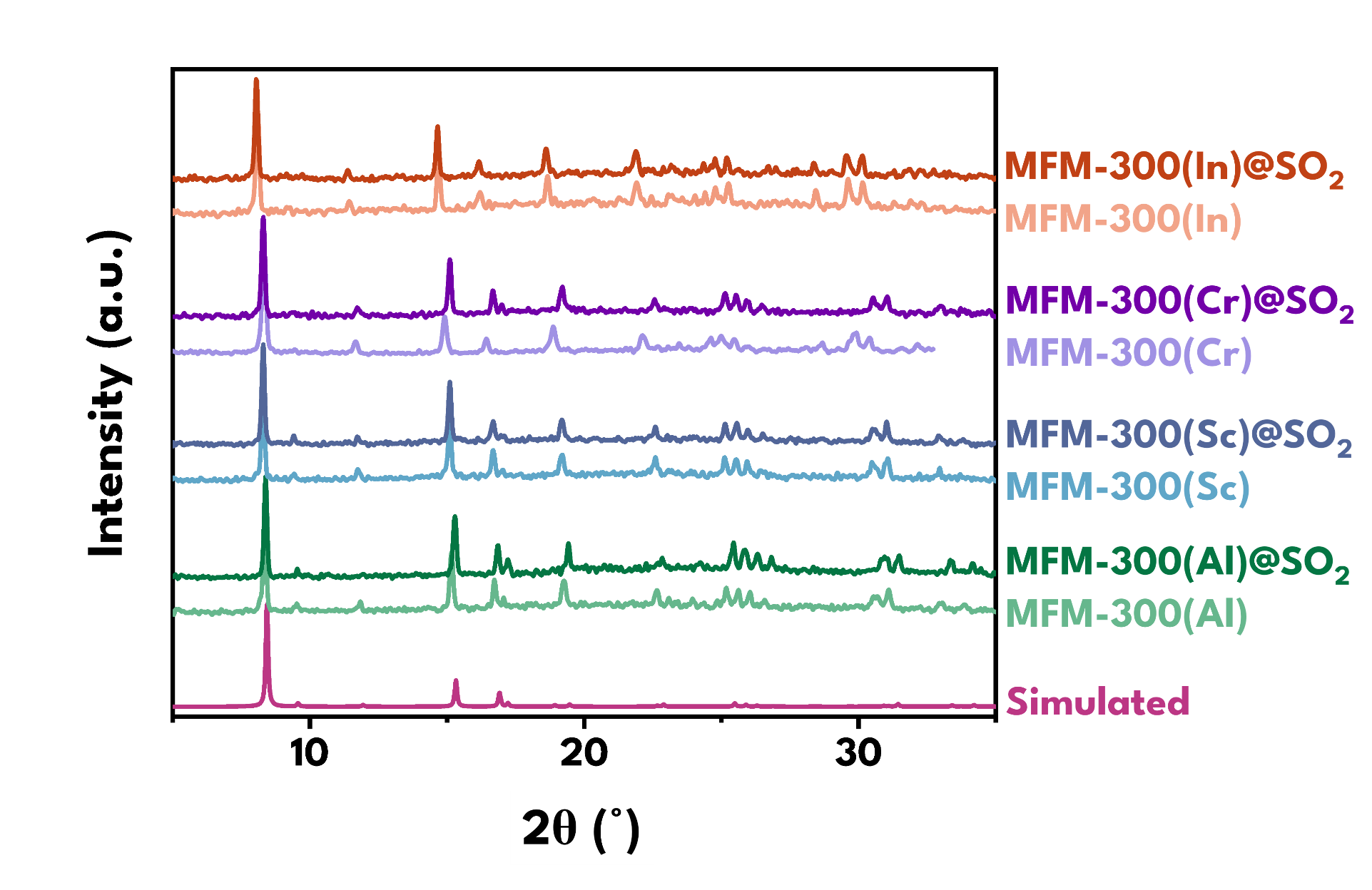
**

**Figure S2.** PXRD patterns of MFM-300(M) materials: simulated (pink), MFM-300(Al) (green), MFM-300(Sc) (blue), MFM-300(Cr) (purple), and MFM-300(In) (orange).

**
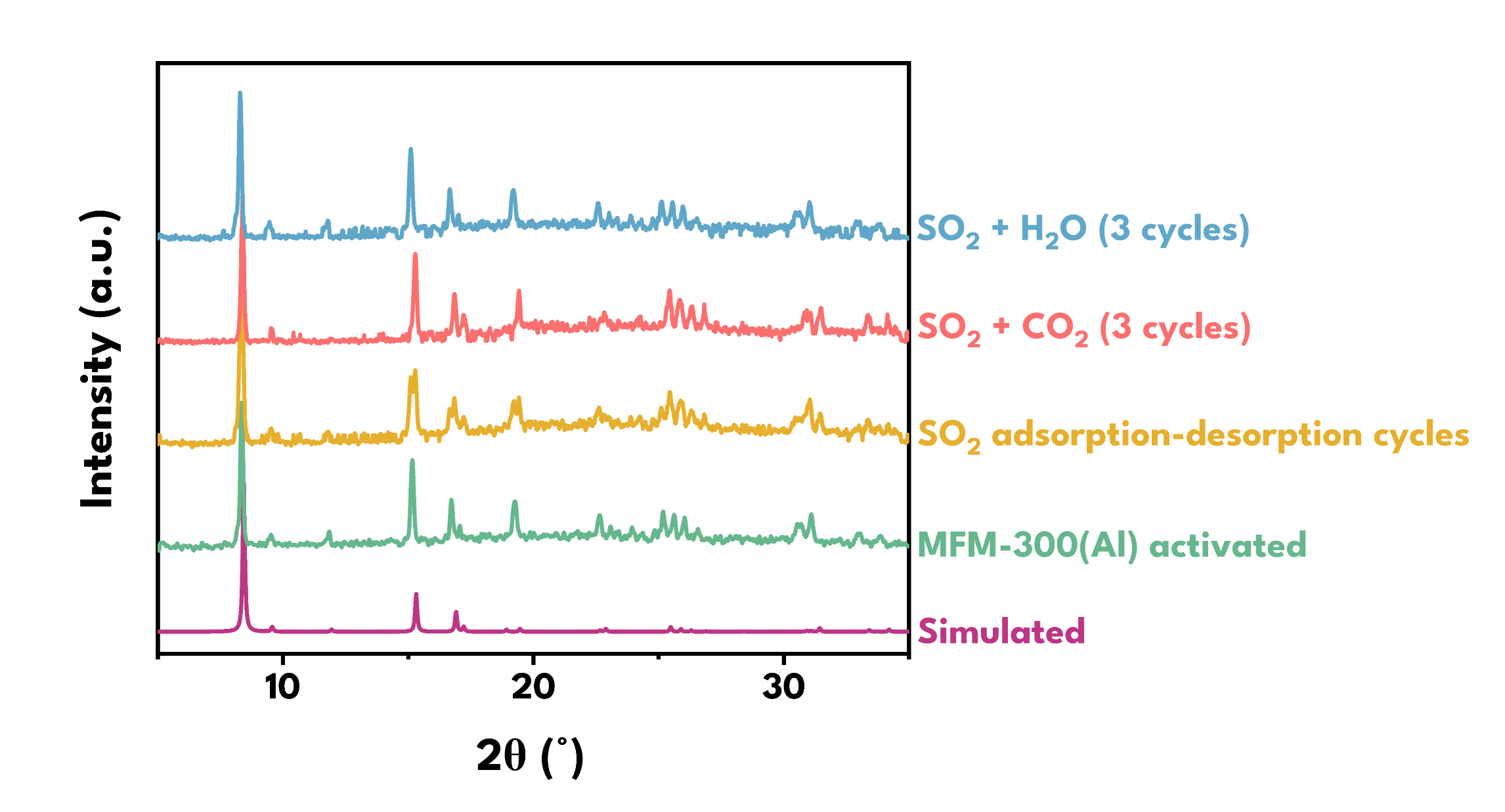
**

**Figure S3.** PXRD patterns of MFM-300(Al) material after five SO_2_ adsorption-desorption cycles (yellow) and after three exposures to the binary mixtures SO_2_ + CO_2_ (coral) and SO_2_ + H_2_O (blue).

**
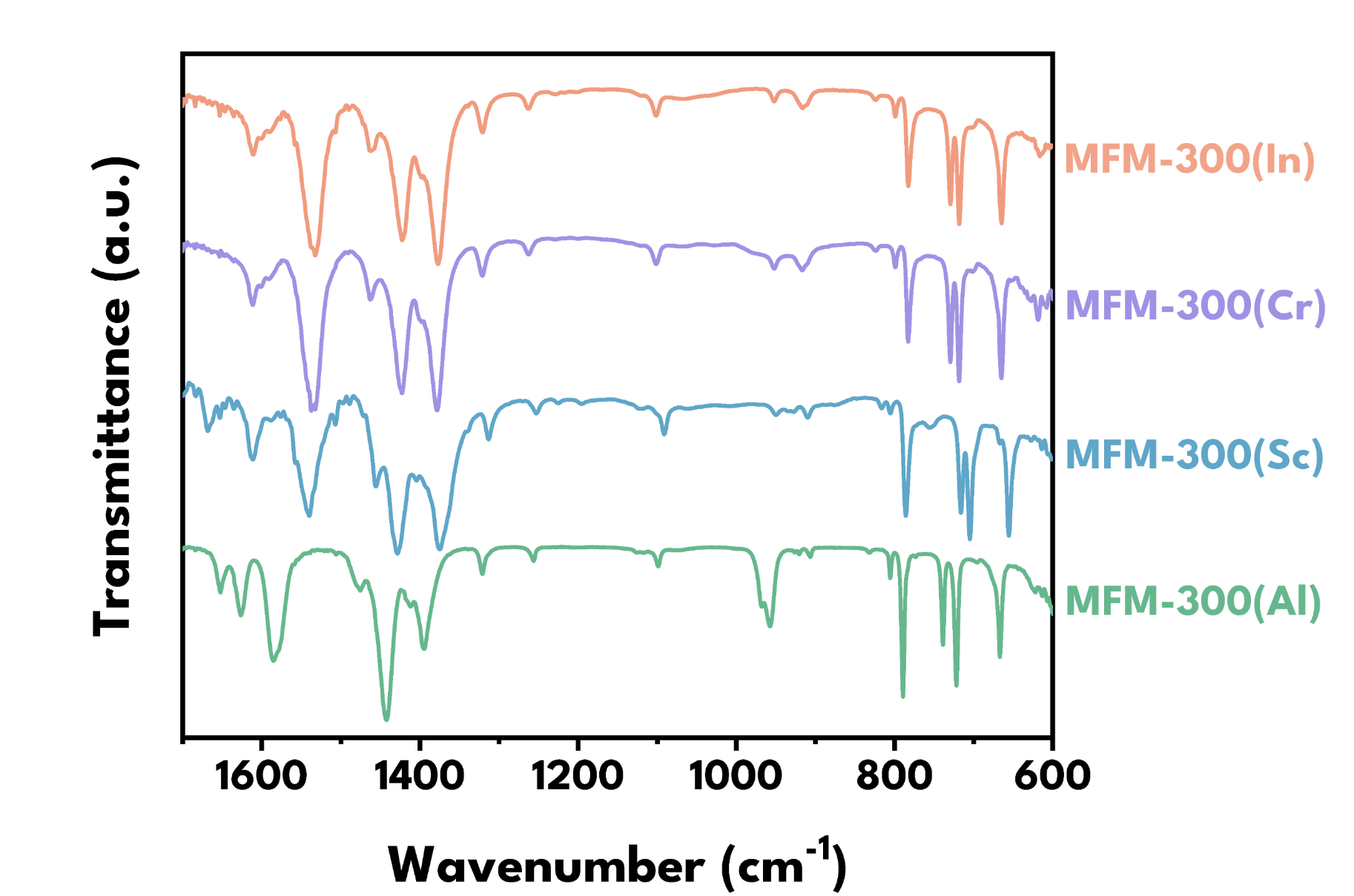
**

**Figure S4.** FTIR spectra of MFM-300(M) materials in the 1700-600 cm^-1^ region: MFM-300(Al) (green), MFM-300(Sc) (blue), MFM-300(Cr) (purple), and MFM-300(In) (orange).

**
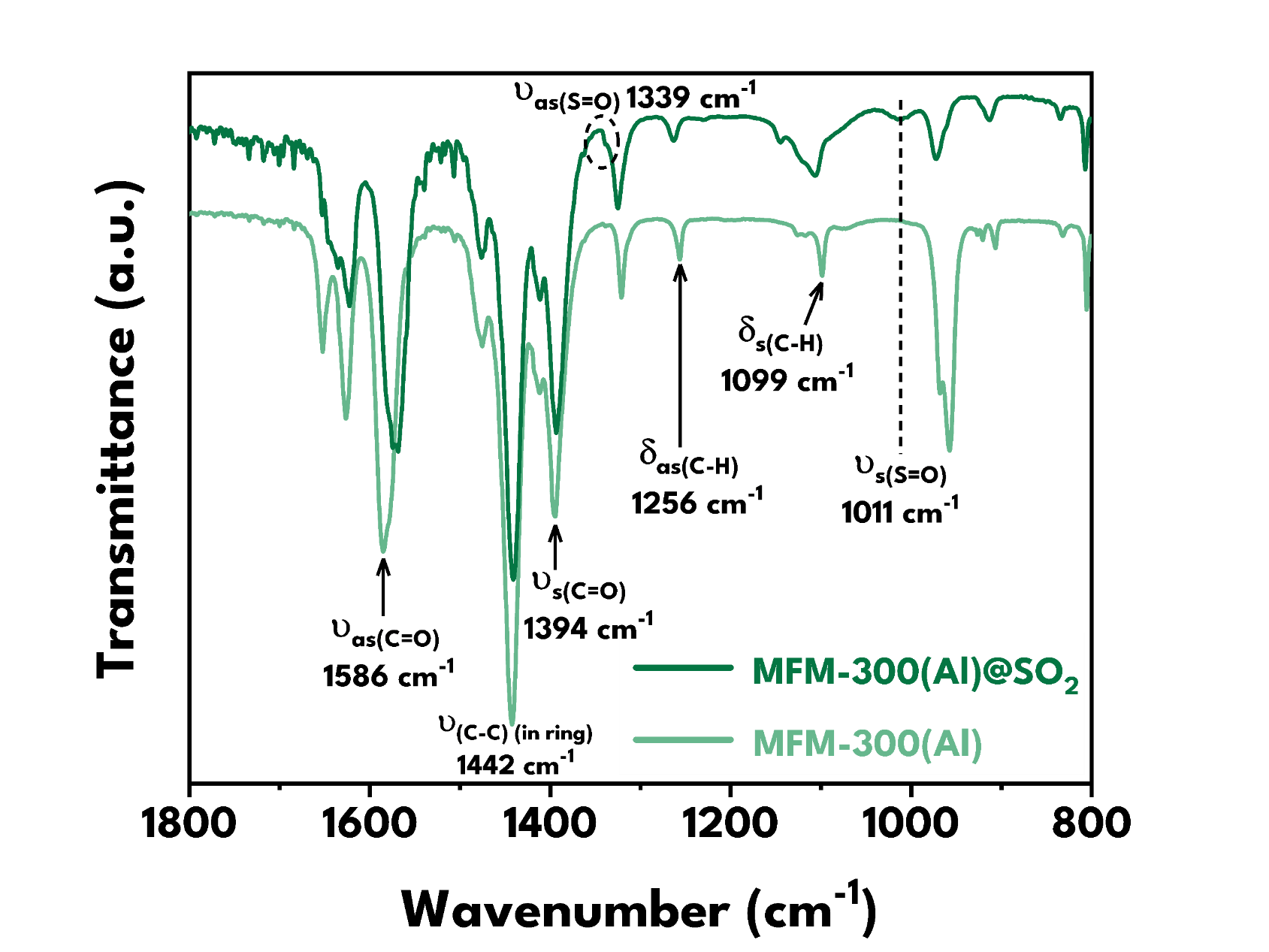
**

**Figure S5.** FTIR spectra of MFM-300(Al) material: activated (light green) and saturated with SO_2_ (dark green).

**
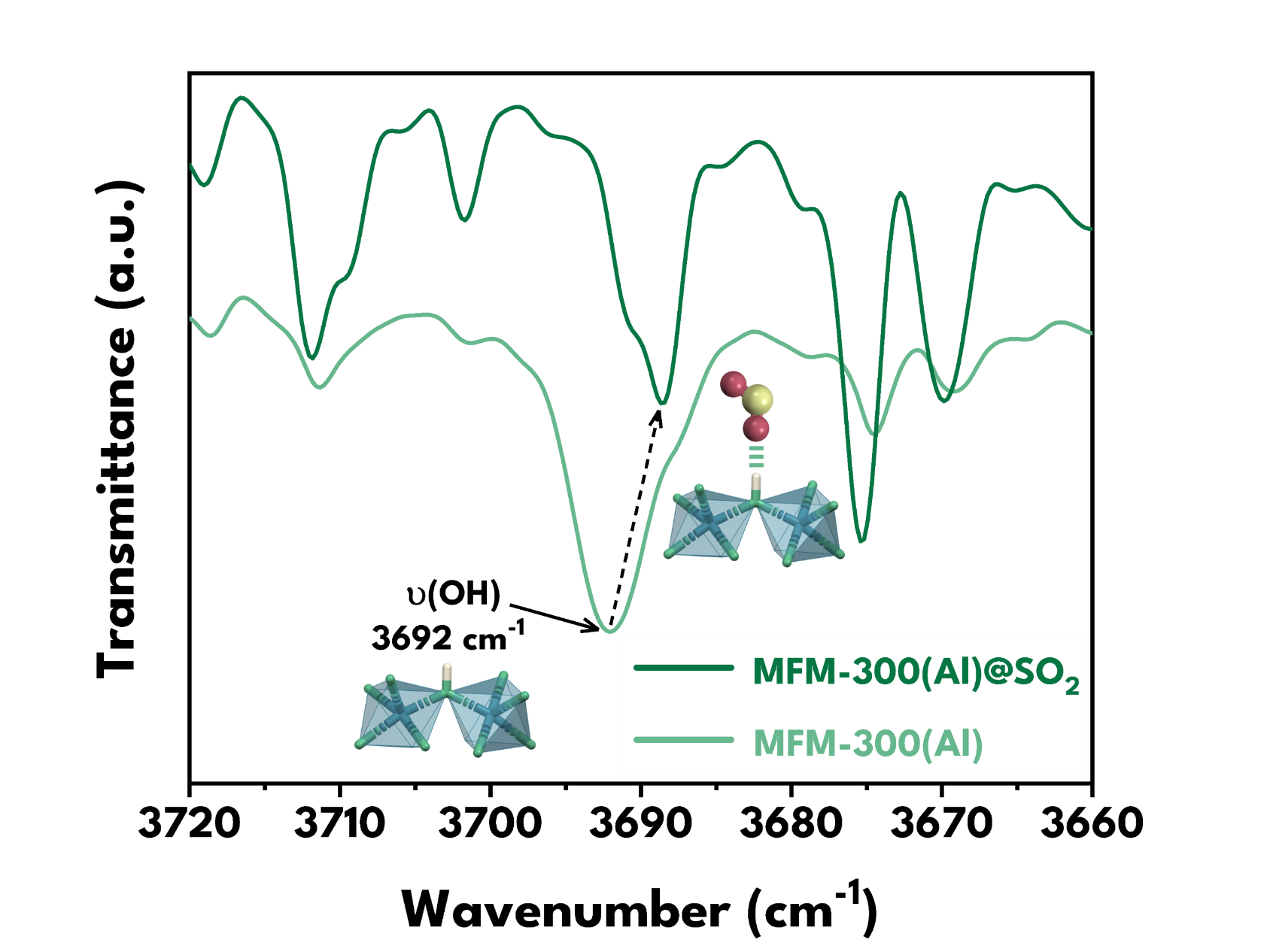
**

**Figure S6.** FTIR spectra in 3600 cm^-1^ of MFM-300(Al) material: activated (light green) and saturated with SO_2_ (dark green).

**
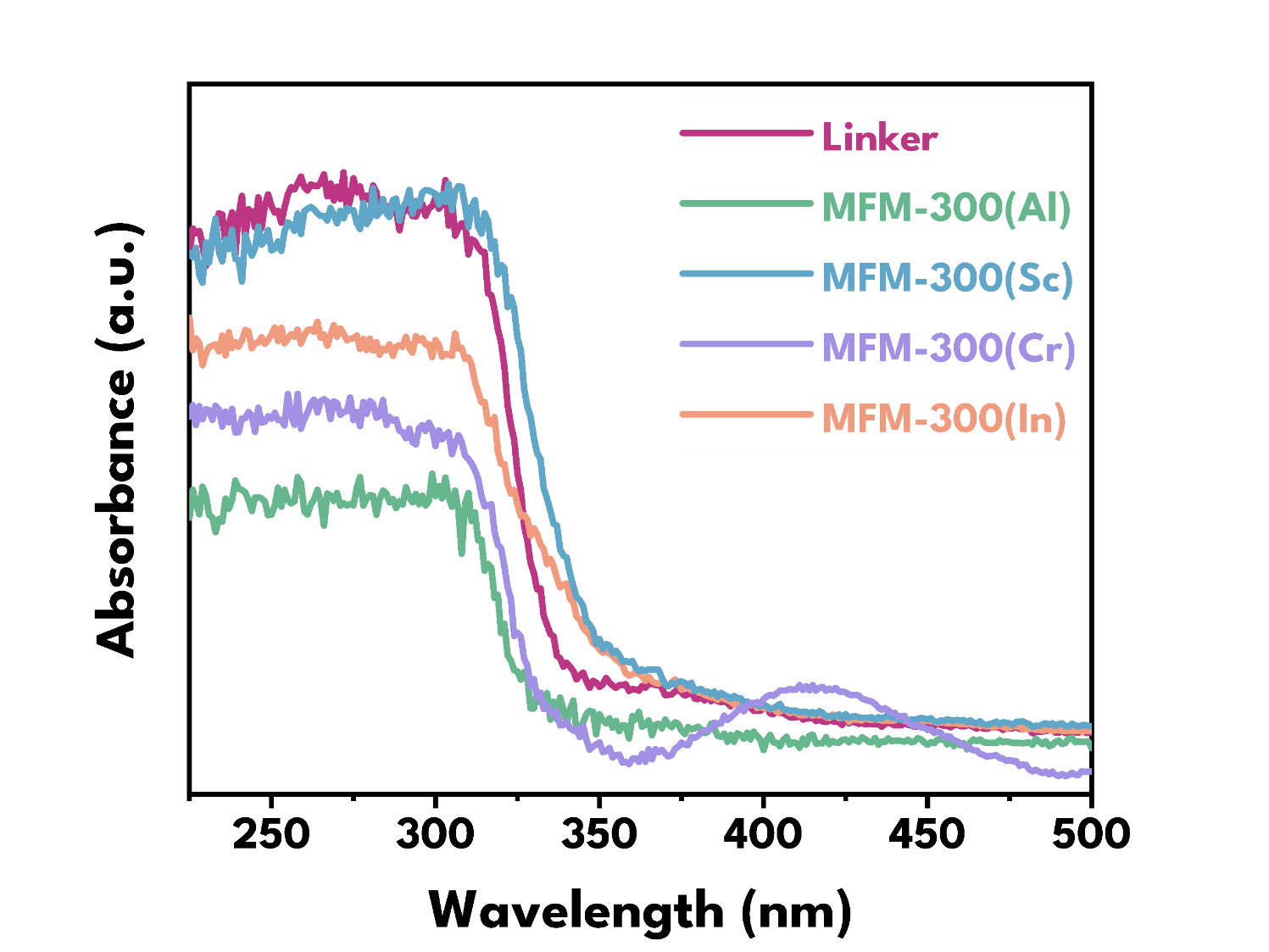
**

**Figure S7.** Solid-state UV-vis spectra of BPTC⁴^-^ ligand (pink), MFM-300(Al) (green), MFM-300(Sc) (blue), MFM-300(Cr) (purple), and MFM-300(In) (orange).


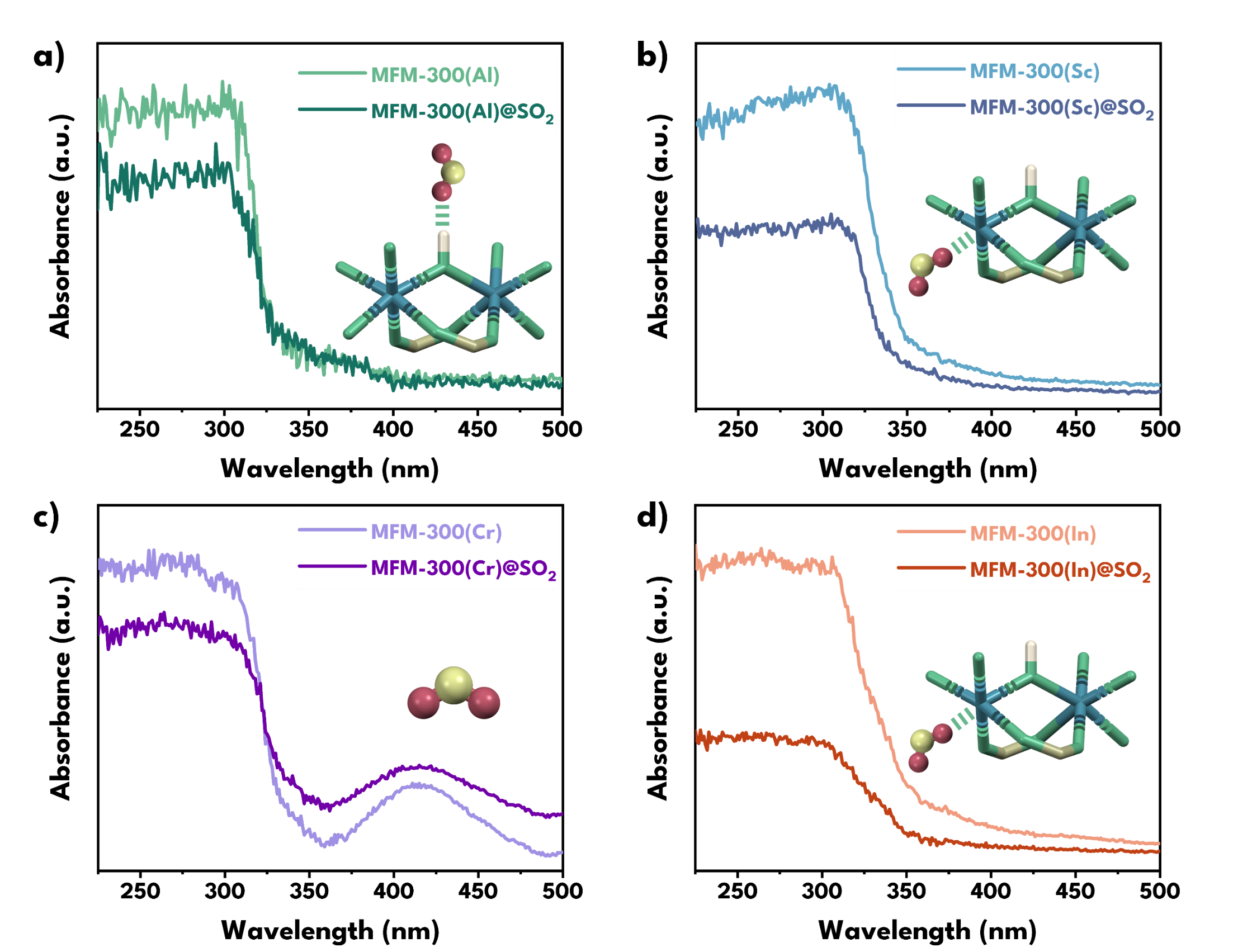


**Figure S8.** Comparison of the solid-state UV-vis spectra of MFM-300(Al) (green), MFM-300(Sc) (blue), MFM-300(Cr) (purple), and MFM-300(In) (orange) before and after SO_2_ exposure.

**S3. Determination of the number of adsorbed molecules per unit cell**

To estimate the number of SO_2_ molecules adsorbed per unit cell on the MFM-300(M) materials at 1 bar, the following relationship is used:

$$\frac{SO_{2} molecules}{Unit Cell}=\frac{{mol}_{SO_{2}}}{g_{sample}}\cdot\frac{g_{sample}}{mol_{sample}}\cdot\frac{formula units}{Unit Cell} (1)$$

Where $\frac{{mol}_{SO_{2}}}{g_{sample}}$ represents the amount of SO_2_ adsorbed at 1 bar, obtained from the adsorption isotherms (Table S1). $\frac{g_{sample}}{mol_{sample}}$ is the molecular weight of the material per unit formula calculated from the molecular formula of the material (Table S1). Finally, the number of formula units per unit cell (Z) in this family of materials (there is one).

**Table S1.** Summary of molecular formulae, molar masses and SO_2_ uptake capacities of the MFM-300(M) materials at 1 bar.

| **Material** | **Molecular formula** | **Molar mass**  **(g mol^-1^)** | **SO_2_ capture (mmol g^-1^)** | **Ref** |
| --- | --- | --- | --- | --- |
| MFM-300(Al) | [Al_2_(OH)_2_(C_16_O_8_H_6_)] | 406.20 | 8.1 | ^[1]^ |
| MFM-300(Sc) | [Sc_2_(OH)_2_(C_16_O_8_H_6_)] | 489.87 | 8.3 | ^[2]^ |
| MFM-300(Cr) | [Cr_2_(OH)_2_(C_16_O_8_H_6_)] | 464.22 | 8.6 | ^[3]^ |
| MFM-300(In) | [In_2_(OH)_2_(C_16_O_8_H_6_)] | 450.14 | 9.4 | ^[4]^ |

The following values are obtained:

$$\frac{SO_{2} molecules}{MFM-300\left( Al \right) unit cell}=\frac{0.0081 mol}{g}\cdot\frac{406.2 g}{mol}\cdot1= 3.29$$

$$\frac{SO_{2} molecules}{MFM-300\left( Cr \right) unit cell}=\frac{0.00859 mol}{g}\cdot\frac{464.22 g}{mol}\cdot1= 3.59$$

$$\frac{SO_{2} molecules}{MFM-300\left( Sc \right) unit cell}=\frac{0.0094 mol}{g}\cdot\frac{450.14 g}{mol}\cdot1= 4.23$$

$$\frac{SO_{2} molecules}{MFM-300\left( In \right) unit cell}=\frac{0.0083 mol}{g}\cdot\frac{589.87 g}{mol}\cdot1= 4.89$$

**S4. Tauc plots for the determination of the energies between HOMO-LUMO orbitals by direct method**

The determination of the energy between the HOMO-LUMO orbitals of the ligand, as well as of the activated and SO_2_-saturated MFM-300(M) materials, were performed by constructing Tauc plots using solid-state UV-visible spectroscopy data.^[5]^ The Tauc plots, shown in Figure S8, were used to assess the optical bandgap assuming a direct allowed transition, based on analyses of the absorption edge.

The following relationship was used for this assessment:

Direct transition: $\left( \alpha h\upsilon\right)^{2}\propto(h\upsilon-E_{gap}) (2)$

Where α is the absorption coefficient, $h\upsilon$ is the photon energy, and E_gap_ represents the HOMO-LUMO energy gap. By extrapolating the linear region of the Tauc plot to α=0, the E_gap_ value for each transition type is obtained.

**Table S2.** HOMO-LUMO energy values considering indirect transitions calculated from the Tauc method for the BPTC⁴^-^ ligand, and the activated and SO_2_-saturated MFM-300(M) materials.

| **Material** | **Activated** | **SO_2_-saturated** |
| --- | --- | --- |
| H_4_BPTC | 3.76 eV | --- |
| MFM-300(Al) | 3.83 eV | 3.82 eV |
| MFM-300(Sc) | 3.71 eV | 3.67 eV |
| MFM-300(In) | 3.80 eV | 3.71 eV |


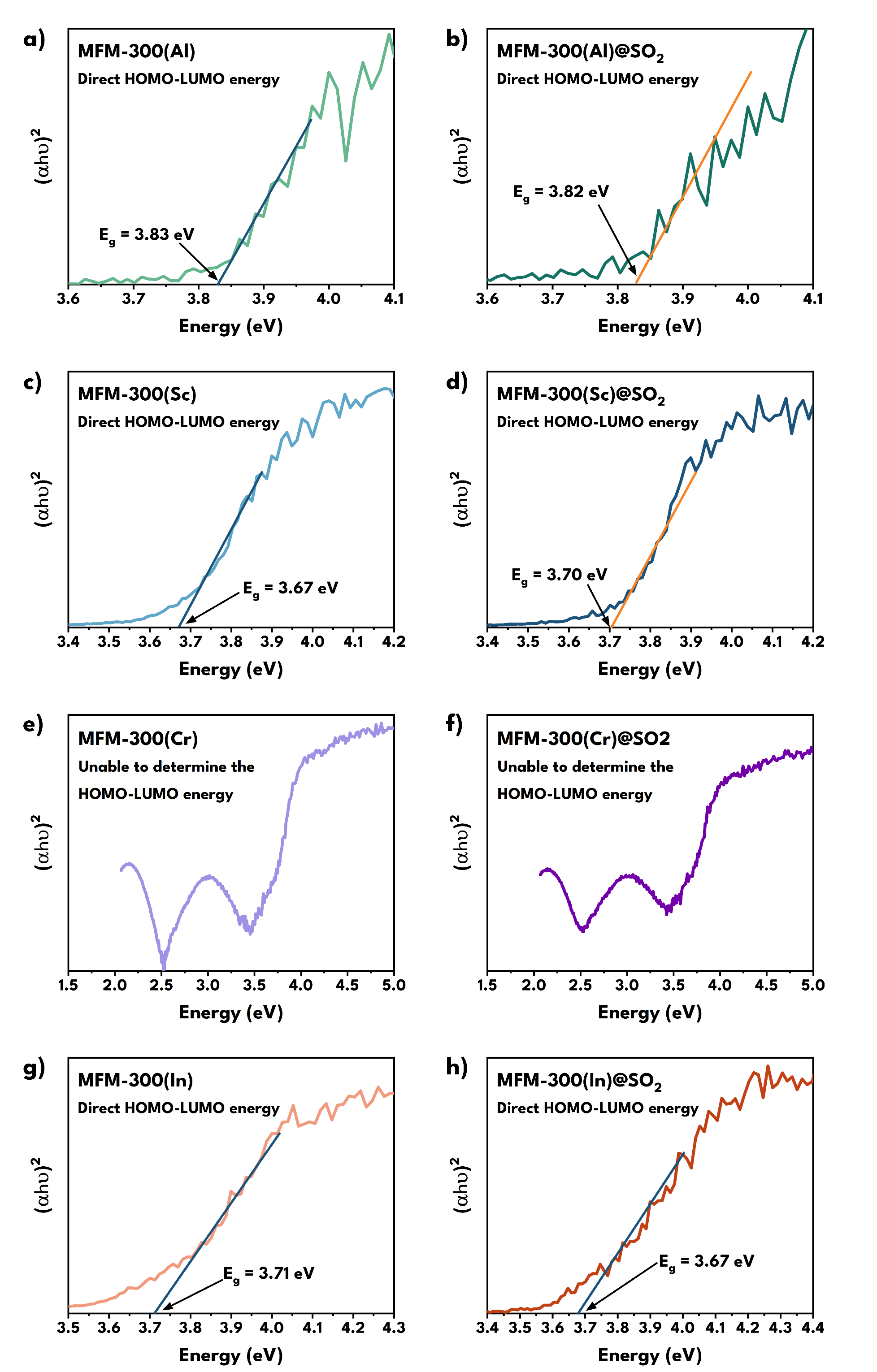


**Figure S9.** Tauc plots considering direct transitions MFM-300(Al) (green), MFM-300(Sc) (blue), MFM-300(Cr) (purple), and MFM-300(In) (orange).

**S5.** **Computational methods**

All Density functional theory (DFT) calculations were performed using the projector augmented-wave (PAW) method, as implemented in the VASP (Vienna Ab initio Simulation Package) code.^[6–9]^ The exchange-correlation energy was calculated using the generalized gradient approximation (GGA) along with the Perdew–Burke–Ernzerhof (PBE) functional.^[10]^ The plane-wave function basis was expanded with a cut-off of 500 eV and employed a gamma 1 × 1 × 1 *k*-point grid for the geometry optimization procedures due to the large size systems.

All structures underwent full relaxation using Hellmann-Feynman forces, with a convergence criterion of less than 1 × 10^−2^ eV Å^−1^, while the energy threshold was established at 1 × 10^−5^ eV. Given that standard DFT lacks a proper description of weak dispersion forces, structural optimizations and total energy calculations were re-evaluated using Grimme’s DFT-D3 dispersion correction.^[11]^ Furthermore, the adsorption energy (E_ads_) was calculated according to the following equation:

E_ads_ = E_total_ - [E_MFM-300(M)_ + E_SO2_] (3)

Where E_total_ corresponds to the total energy of the interaction of SO_2_ molecule inside the MFM-300(M) evaluated, E_MFM-300(M)_ refers to the total energy of MOF structure, and E_SO2_ is the electronic energy of SO_2_ molecule isolated. A negative value of E_ads_ indicates that the interaction between the SO_2_ molecule and the MOF surface is more attractive.

In addition, the optical properties were calculated using the SIESTA *ab initio* code,^[12]^ as described by Equation (4):^[13]^

e(w)=e_1_(w)+ie_2_(w) (4)

where the dielectric function considers its tensor nature, i.e., e_1_(w) and e_2_(w), averaged over three polarization vectors along the *x*, *y*, and *z* directions. Finally, molecular dynamics (MD) simulations were performed in the *NVT* ensemble at a temperature of 300 °C. The simulations were run for 5 ps with a time step of 2.5 fs. All MD calculations were carried out using the SIESTA ab initio code.^[12]^

**Table S3.** Geometrical parameters (Å) of the fully relaxed MFM-300(M). All values were calculated at the DFT+D3 level of theory.

| **Systems** | **a=b (Å)** | **c (Å)** |
| --- | --- | --- |
| MFM-300(Al) | 15.48 | 12.28 |
| MFM-300(Sc) | 15.69 | 12.80 |
| MFM-300(Cr) | 14.91 | 12.06 |
| MFM-300(In) | 16.03 | 13.28 |

**Table S4.** Comparsion between M-O bond distances in the MFM-300(M=Al, Sc, Cr, In) calculated by DFT+D3 calculations and reported experimental data.

| **Material** | **Theorical**  **M-O_carboxilate_ (Å)** | **Reported**  **M-O_carboxilate_  (Å)** | **Theorical**  **M-O_M-OH_ (Å)** | **Reported**  **M-O_M-OH_ (Å)** |
| --- | --- | --- | --- | --- |
| MFM-300(Al) | 2.00 | 1.93^[26]^ | 1.94 | 1.93^[14]^ |
| MFM-300(Sc) | 2.15 | 2.02^[25]^ | 2.10 | 1.95^[15]^ |
| MFM-300(Cr) | 1.92 | 2.11^[35]^ | 1.97 | 2.05^[16]^ |
| MFM-300(In) | 2.28 | 2.15^[36]^ | 2.18 | 2.06^[17]^ |

**Table S5.** M-SO_2_ bond distances and energy adsorption values for the MFM-300(M=Al, Sc, Cr, In) calculated by DFT+D3 calculations.

| **Material** | **M-SO_2_ (Å)** | **E_ads_ (eV)** |
| --- | --- | --- |
| SO_2_/MFM-300(Al) | 4.17 | -0.23 |
| SO_2_/MFM-300(Sc) | 3.48 | -0.12 |
| SO_2_/MFM-300(Cr) | 3.91 | -0.27 |
| SO_2_/MFM-300(In) | 3.39 | -0.28 |


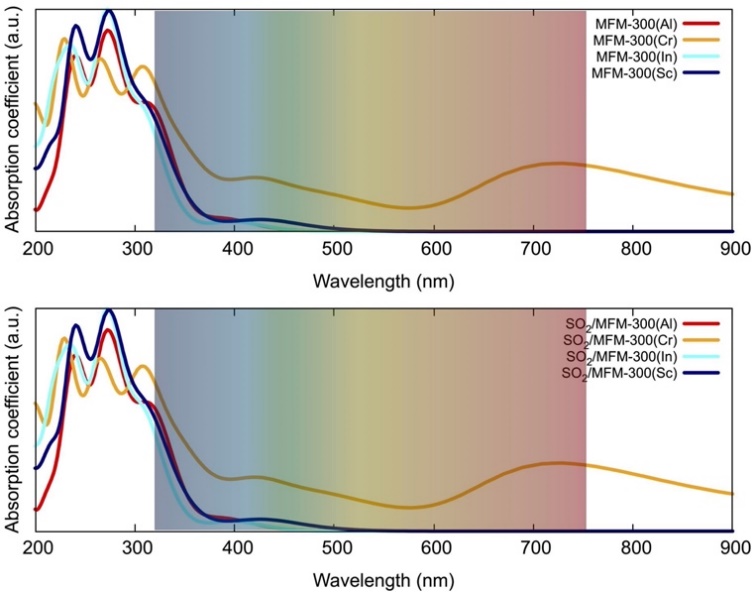


**Figure S10**. Theoretical UV-Vis absorption spectra for MFM-300(M=Al, Cr, In, Sc) after and before the SO_2_ interaction.

**S6. Fluorescence spectroscopy**


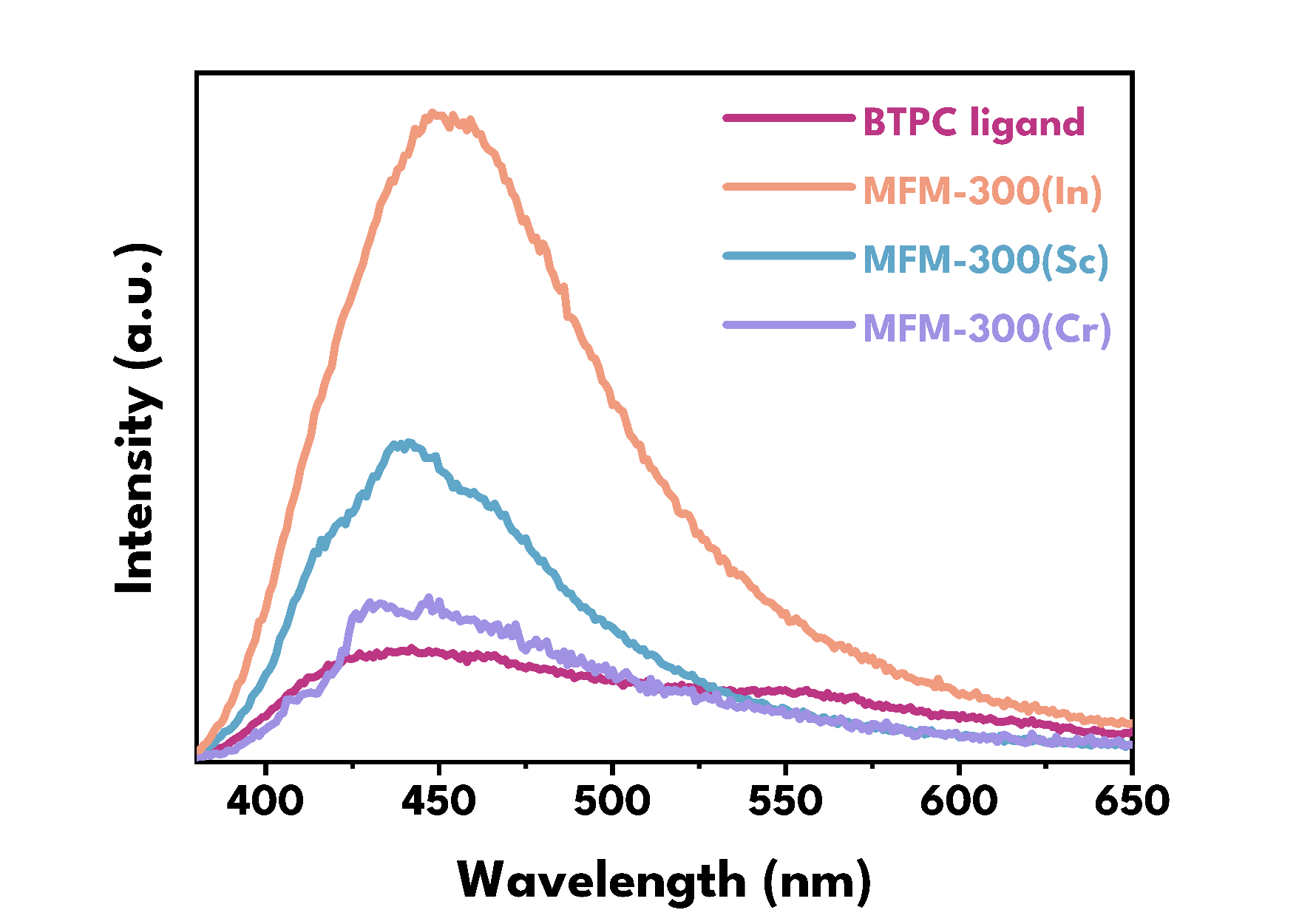


**Figure S11.** Solid-state emission spectra of BPTC⁴^-^ ligand (pink), MFM-300(Sc) (blue), MFM-300(Cr) (purple), and MFM-300(In) (orange).


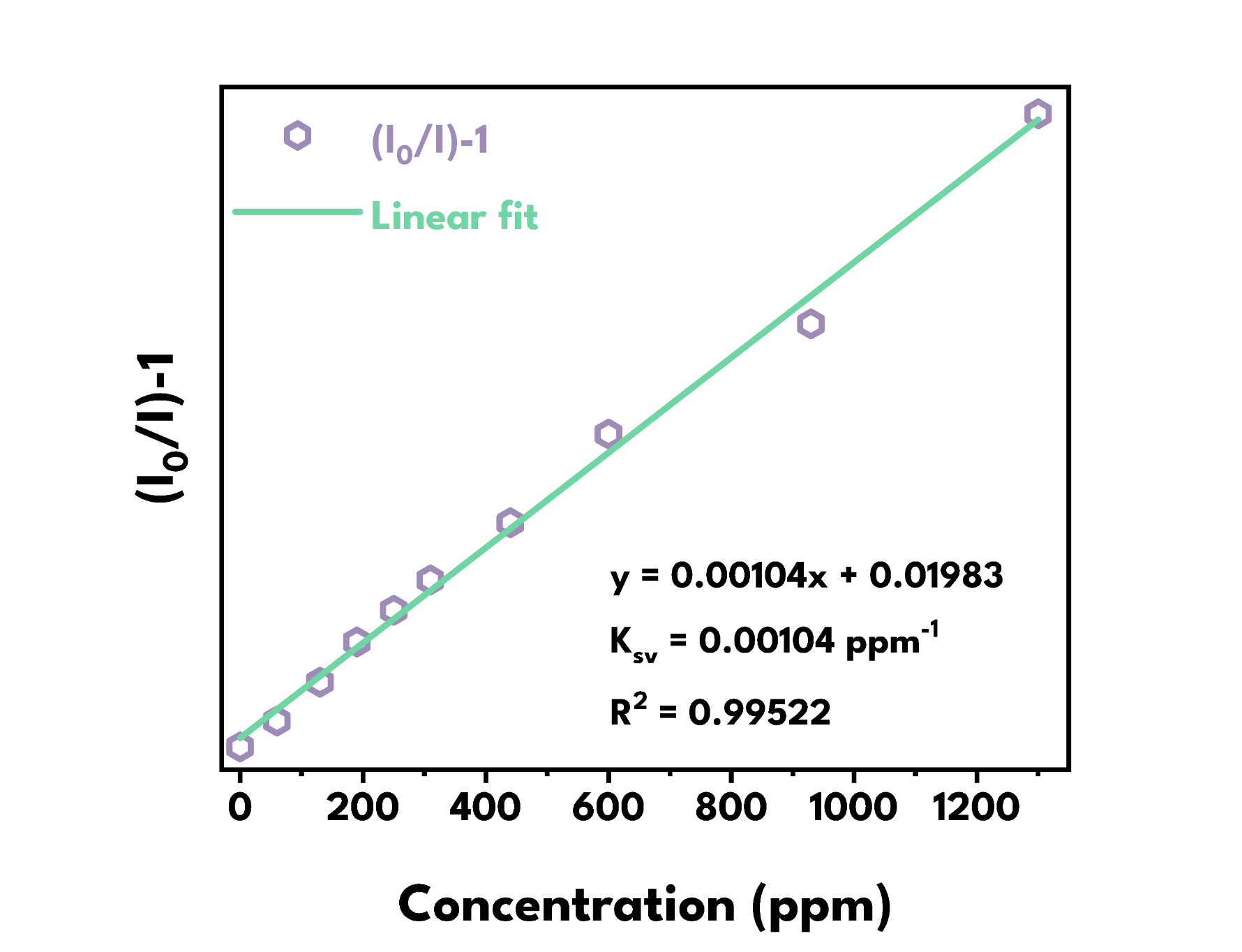


**Figure S12.** Stern-Volmer (SV) plot for MFM-300(Al).


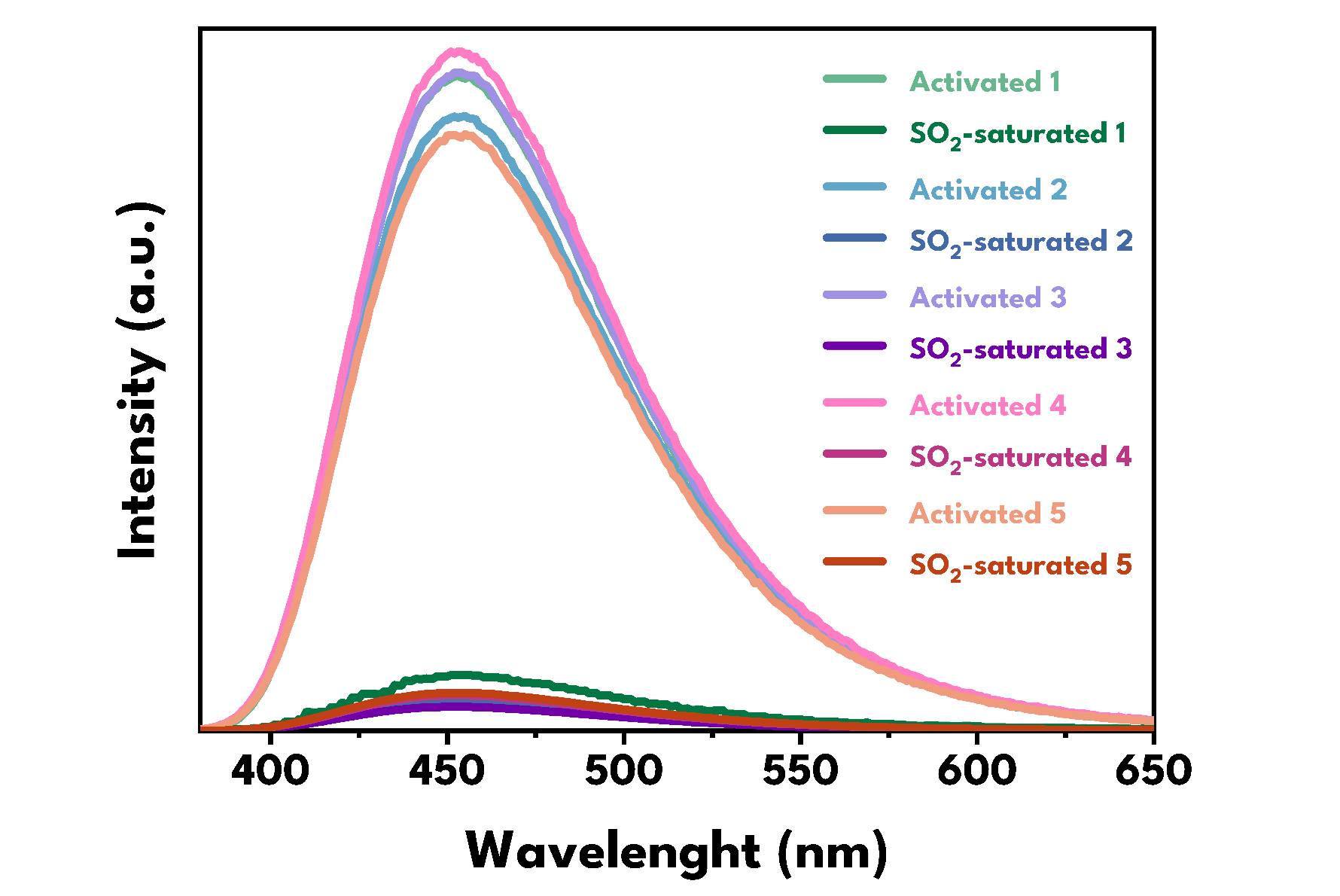


**Figure S13.** Comparison of solid-state emission spectra of MFM-300(Al) exposed to five SO_2_ adsorption-desorption cycles.


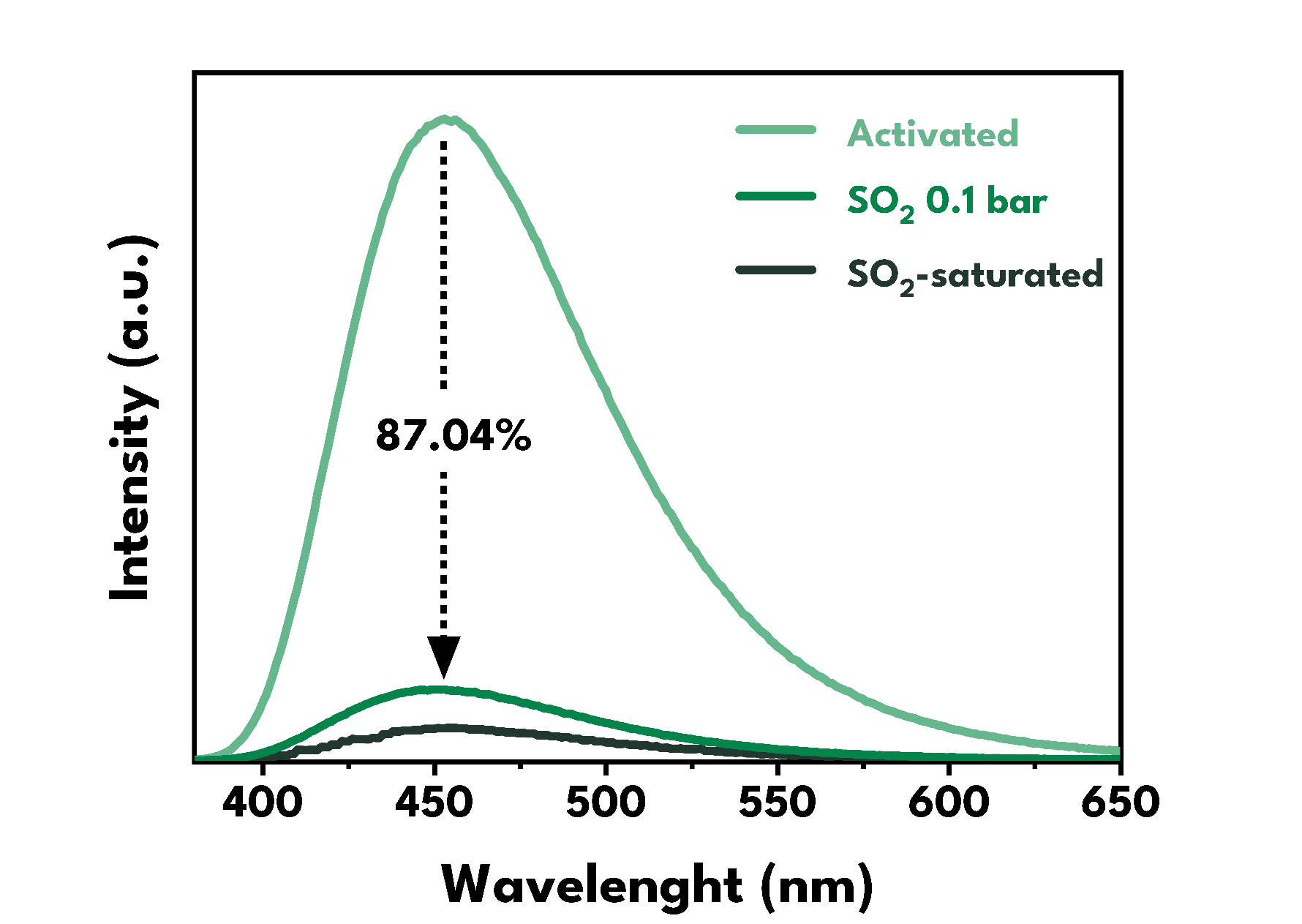


**Figure S14.** Comparison of solid-state emission spectra of MFM-300(Al) exposed to 0.1 bar of SO_2_ and SO_2_-saturated.

**S7. TRPL spectroscopy**

Fluorescence lifetimes were determined from the TPRL spectra. Data obtained from the decay spectra were globally fitted in Fluoracle software from Edinburgh Instruments (Figure S15), using a multi-exponential equation (Equation 2) to describe the fluorescence emission decay curve, through a reconvolution analysis with the instrument response function (IRF) measured under the same experimental conditions:^[18]^

$$R\left( t \right)=B{}_{1}e^{\left( \frac{-t}{\tau_{1}} \right)}+B{}_{2}e^{\left( \frac{-t}{\tau_{2}} \right)}+B{}_{3}e^{\left( \frac{t}{\tau_{3}} \right)}+B{}_{4}e^{\left( \frac{-t}{\tau_{4}} \right)} (5)$$

where R(t) represents the fluorescence intensity as a function of time; B_1_, B_2_, B_3_ and B_4_ are the amplitudes of the respective decay components; and τ_1_, τ_2_, τ_3_ and τ_4_ are the lifetimes of the different components. Additionally, the χ^2^ value is reported.

Here we present as example the graph obtained directly from the Fluoracle software for the MFM-300(Al) spectrum, showing: the complete decay trace with the IRF superimposed in blue, the fit in pink, and the residual panel in green.


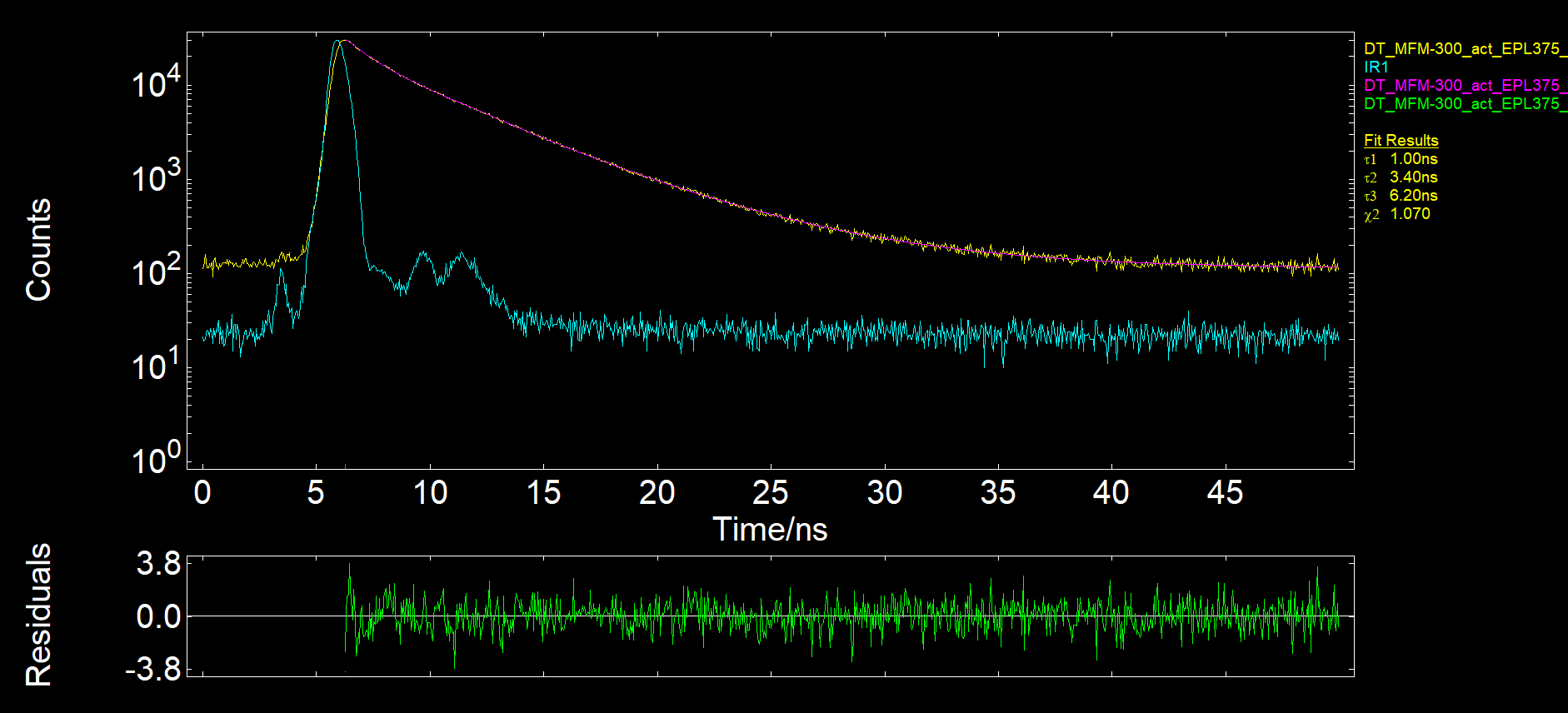


**Figure S15**. Global tri-exponential fit for solid-state activated MFM-300(Al) decay with its corresponding residuals.


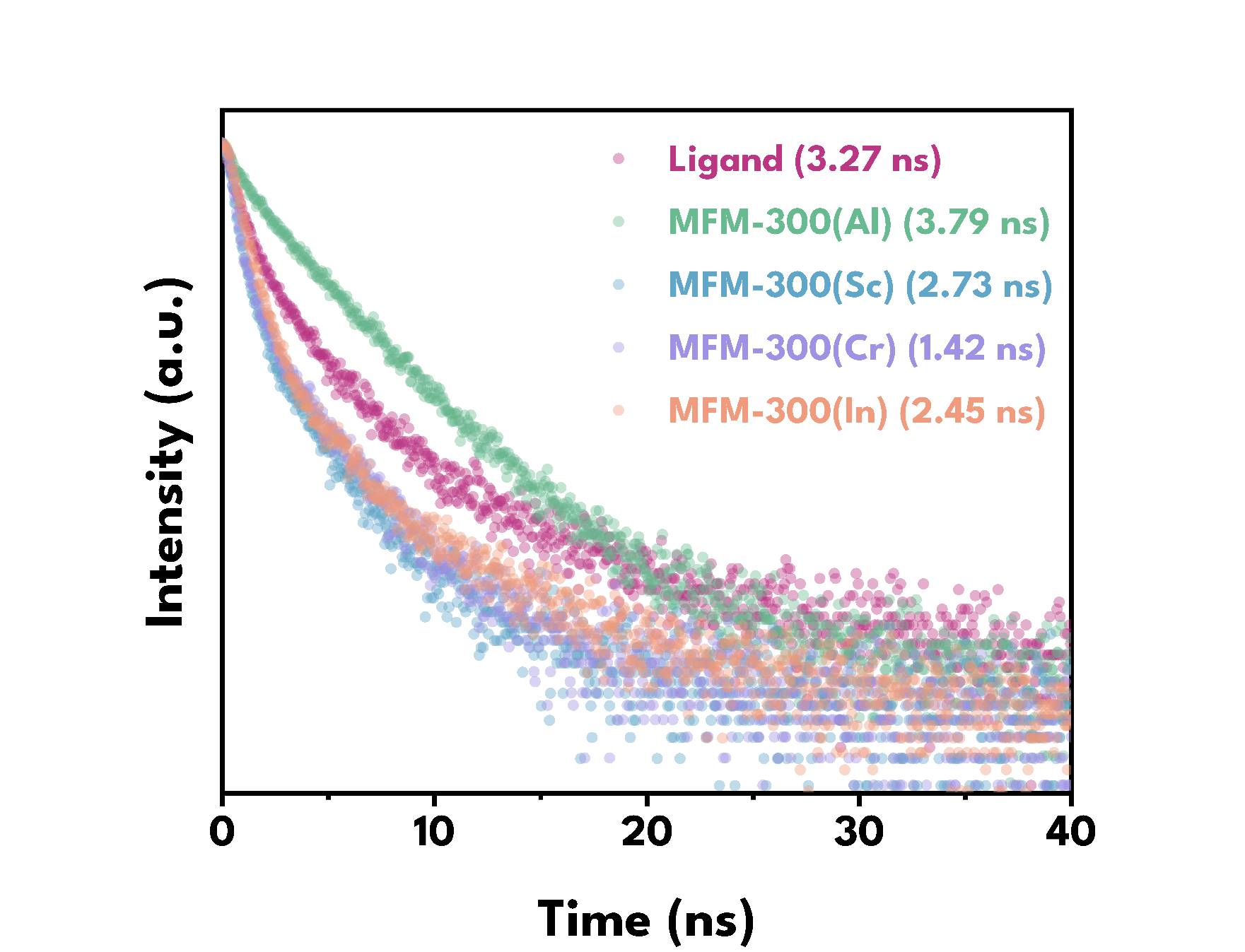


**Figure S16**. Time-resolved photoluminescence decay profiles at λ_em_= 455 nm of solid-state H_4_BPTC ligand and MFM-300(M) materials.


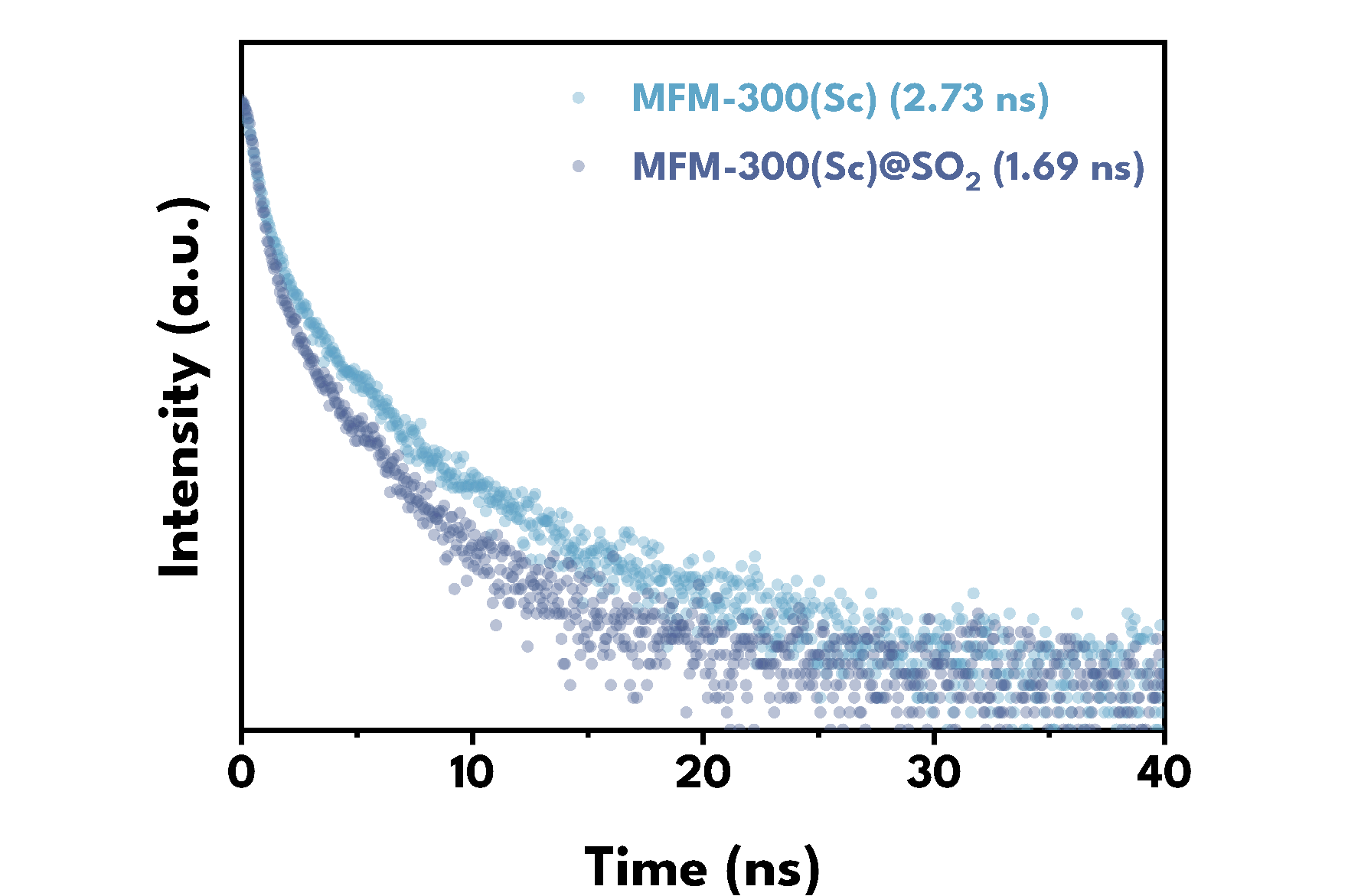


**Figure S17**. Time-resolved photoluminescence decay profile at λ_em_ = 455 nm of solid-state MFM-300(Sc) before and after SO_2_ exposure.


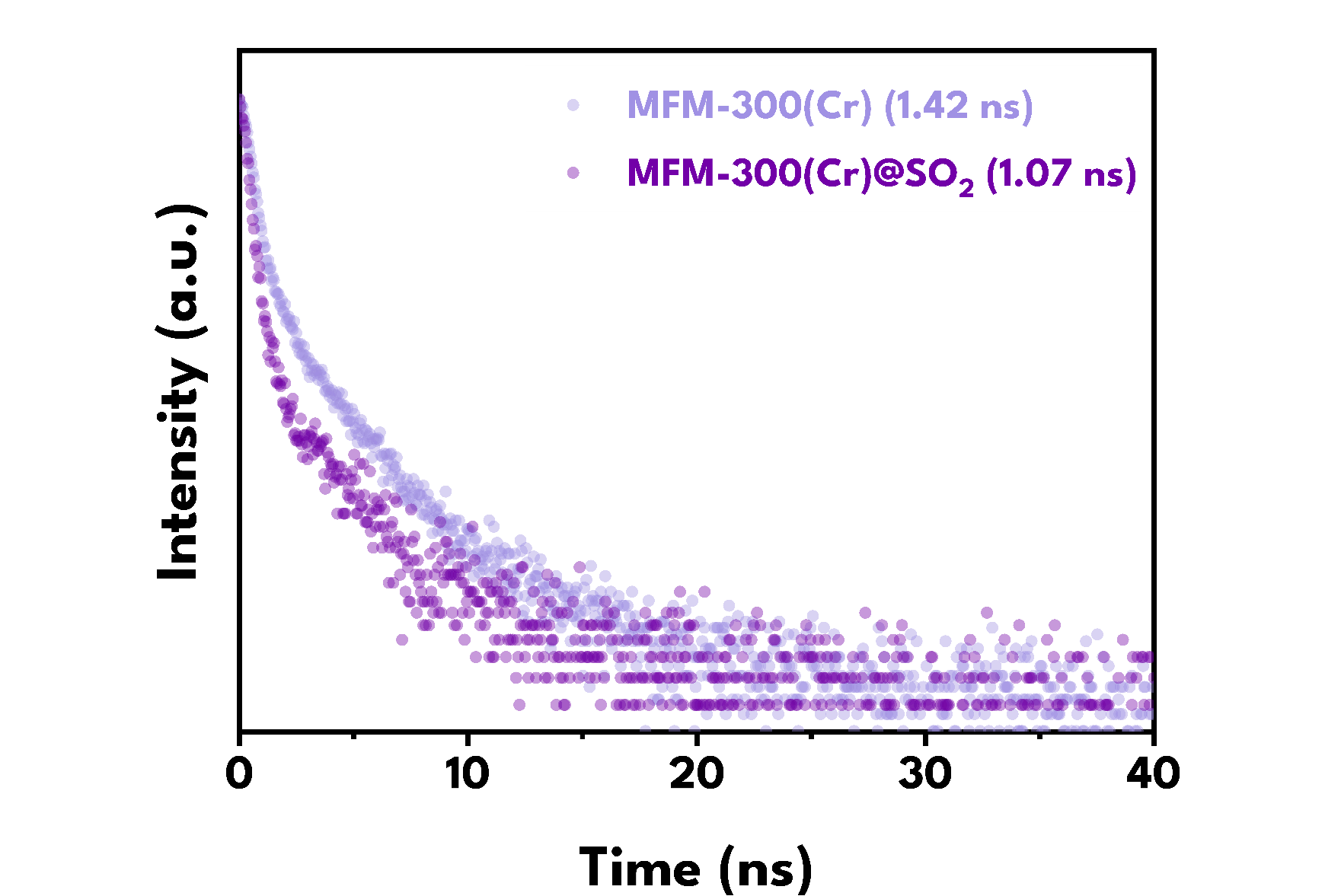


**Figure S18**. Time-resolved photoluminescence decay profile at λ_em_ = 455 nm of solid-state MFM-300(Cr) before and after SO_2_ exposure.


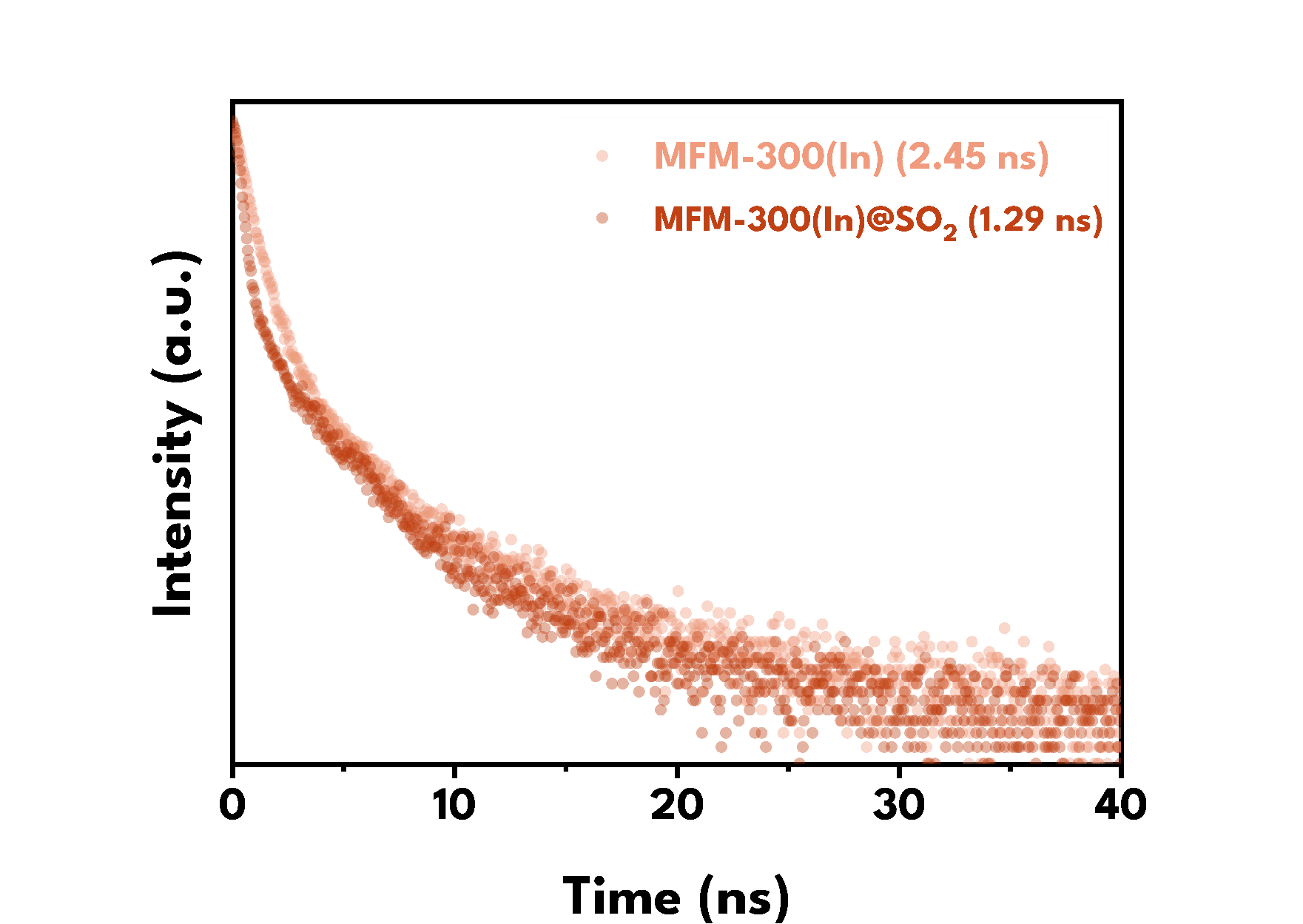


**Figure S19**. Time-resolved photoluminescence decay profile at λ_em_ = 455 nm of solid-state MFM-300(In) before and after SO_2_ exposure.

**Table S6.** Lifetimes of the MFM-300(M) series at λ_em_= 454 nm.

|  | **τ_1_ (ns)** | **a_1_** | **τ_2_ (ns)** | **a_2_** | **τ_3_ (ns)** | **a_3_** | **Lifetime (ns)** | **χ^2^** |
| --- | --- | --- | --- | --- | --- | --- | --- | --- |
| **BPTC** | 0.5693 | 0.2916 | 2.3185 | 0.4768 | 8.6173 | 0.2316 | **3.2672** | 1.0726 |
| **Al** | 1.0026 | 0.1435 | 3.4012 | 0.5939 | 6.2038 | 0.2626 | **3.7919** | 1.0702 |
| **Al@SO_2_** | 0.0910 | 0.5201 | 1.1511 | 0.2449 | 3.2672 | 0.2350 | **1.0853** | 1.2449 |
| **Sc** | 0.4589 | 0.3676 | 1.9419 | 0.4016 | 7.7188 | 0.2308 | **2.7301** | 1.2621 |
| **Sc@SO_2_** | 0.3606 | 0.4583 | 1.5554 | 0.3732 | 5.5921 | 0.1685 | **1.6880** | 1.2020 |
| **Cr** | 0.3066 | 0.5561 | 1.7591 | 0.3081 | 6.4682 | 0.1358 | **1.4204** | 1.2202 |
| **Cr@SO_2_** | 0.2379 | 0.7362 | 1.3627 | 0.1715 | 7.1513 | 0.0923 | **1.0689** | 1.0896 |
| **In** | 0.4178 | 0.4361 | 1.8479 | 0.3518 | 7.6294 | 0.2121 | **2.4505** | 1.1060 |
| **In@SO_2_** | 0.1928 | 0.6566 | 1.4970 | 0.2013 | 6.0434 | 0.1421 | **1.2867** | 1.0129 |

**Table S7.** Lifetimes of the SO_2_-exposed MFM-300(Al) dispersed in THF at λ_em_= 454 nm.

|  | **τ_1_ (ns)** | **a_1_** | **τ_2_ (ns)** | **a_2_** | **τ_3_ (ns)** | **a_3_** | **Lifetime (ns)** | **χ^2^** |
| --- | --- | --- | --- | --- | --- | --- | --- | --- |
| **Pristine** | 1.1854 | 0.0488 | 3.0303 | 0.2925 | 11.4674 | 0.6587 | **8.3831** | 1.1572 |
| **130 ppm** | 0.9638 | 0.0582 | 2.9122 | 0.3176 | 9.2939 | 0.6241 | **6.7802** | 1.1331 |
| **250 ppm** | 1.2267 | 0.1278 | 3.1528 | 0.3285 | 7.9041 | 0.5437 | **5.4899** | 1.0995 |
| **310 ppm** | 1.1320 | 0.1354 | 2.9834 | 0.3692 | 6.8068 | 0.4953 | **4.6215** | 1.0323 |
| **440 ppm** | 1.0294 | 0.1427 | 2.8247 | 0.4099 | 5.7689 | 0.4473 | **3.8852** | 1.1183 |
| **600 ppm** | 1.0271 | 0.2186 | 3.0927 | 0.5788 | 5.4040 | 0.2026 | **3.1094** | 1.0752 |
| **930 ppm** | 0.9815 | 0.3122 | 2.8738 | 0.6643 | 9.4583 | 0.0235 | **2.4378** | 0.9918 |


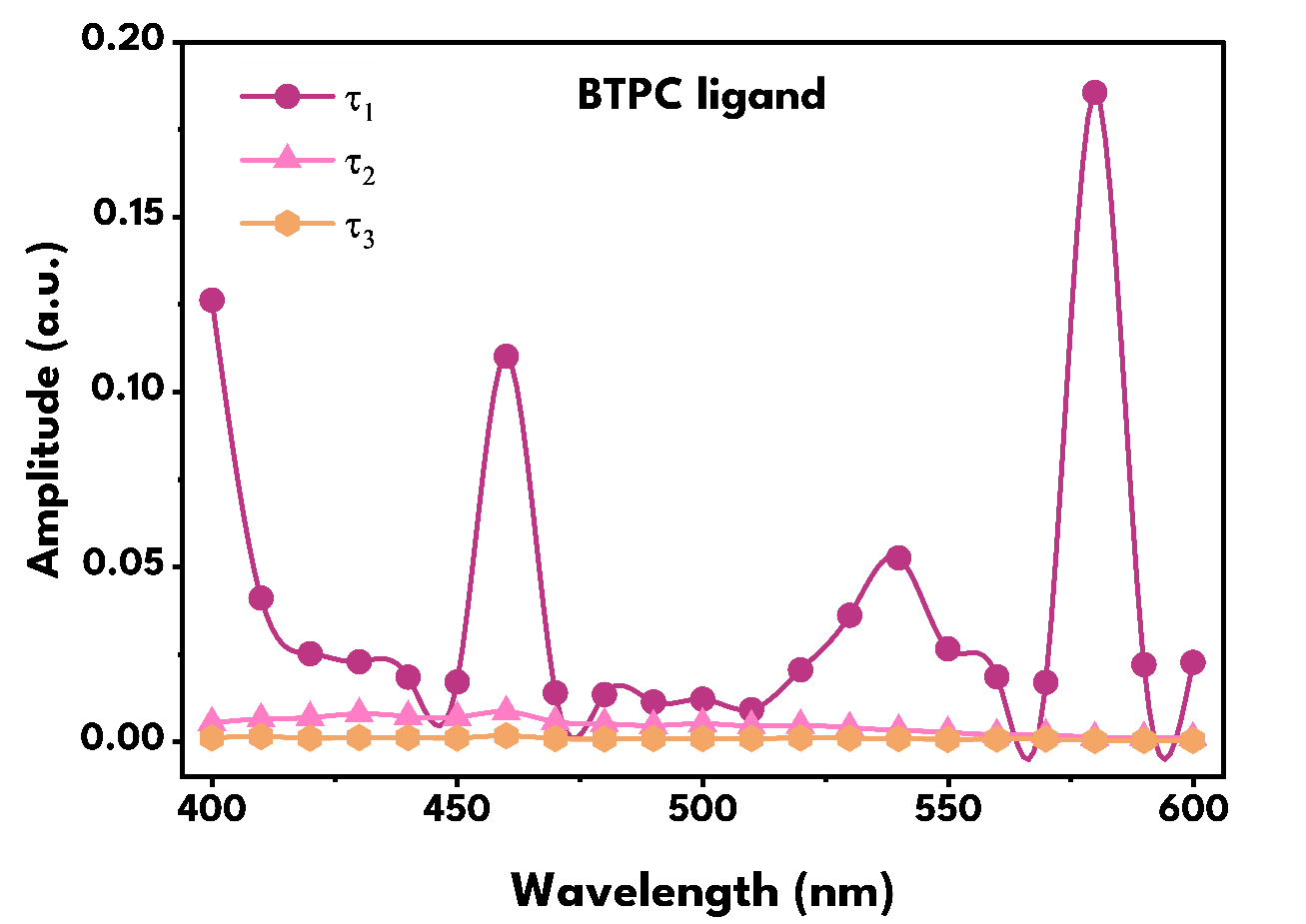


**Figure S20**. DAS of the H_4_BPTC ligand.


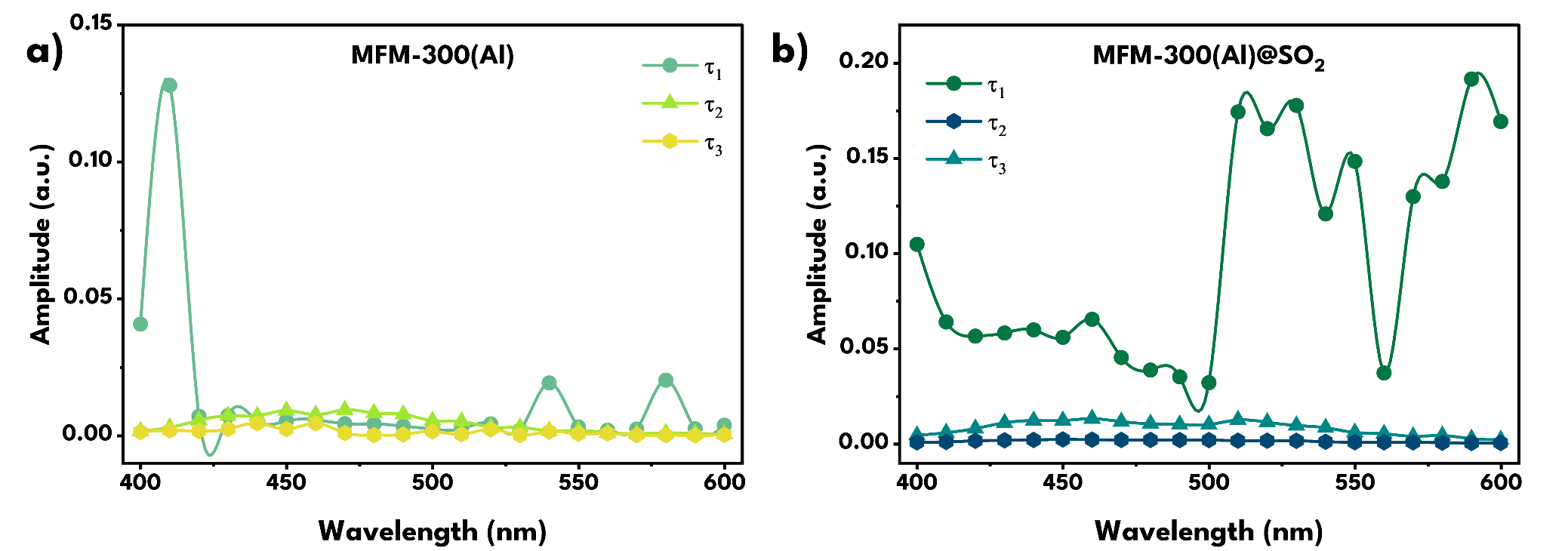


**Figure S21**. DAS of MFM-300(Al) a) before and b) after SO_2_ saturation.


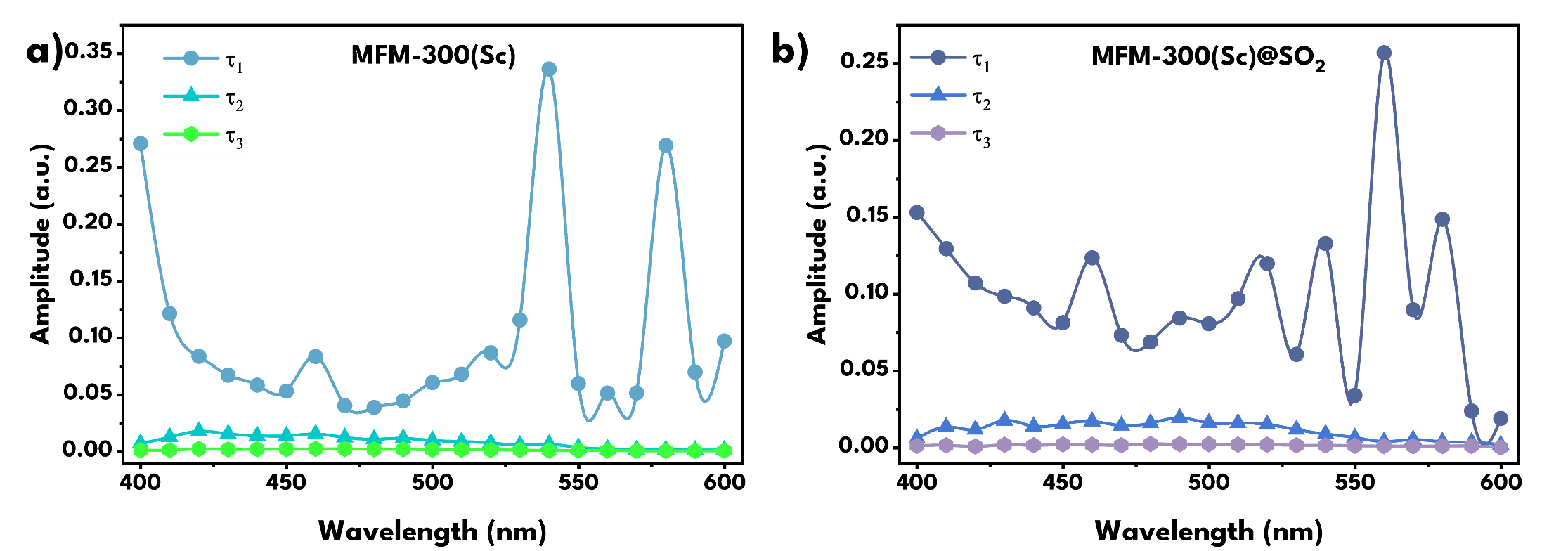


**Figure S22**. DAS of MFM-300(Sc) a) before and b) after SO_2_ saturation.


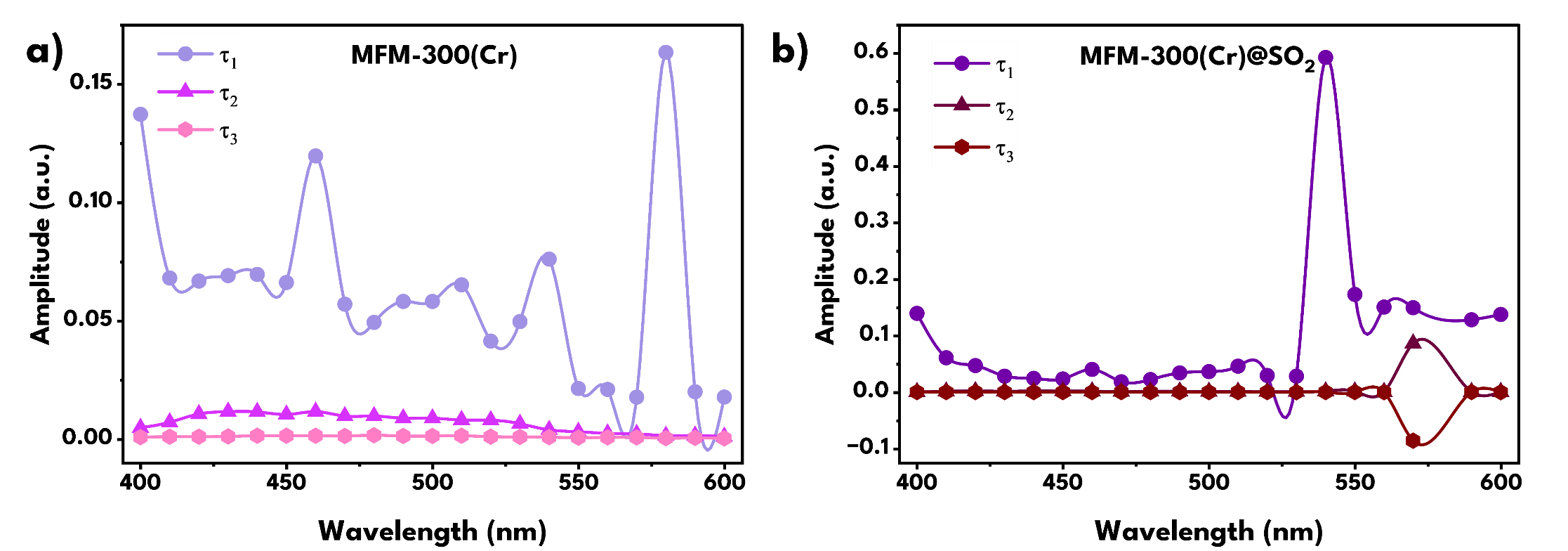


**Figure S23**. DAS of MFM-300(Cr) a) before and b) after SO_2_ saturation.


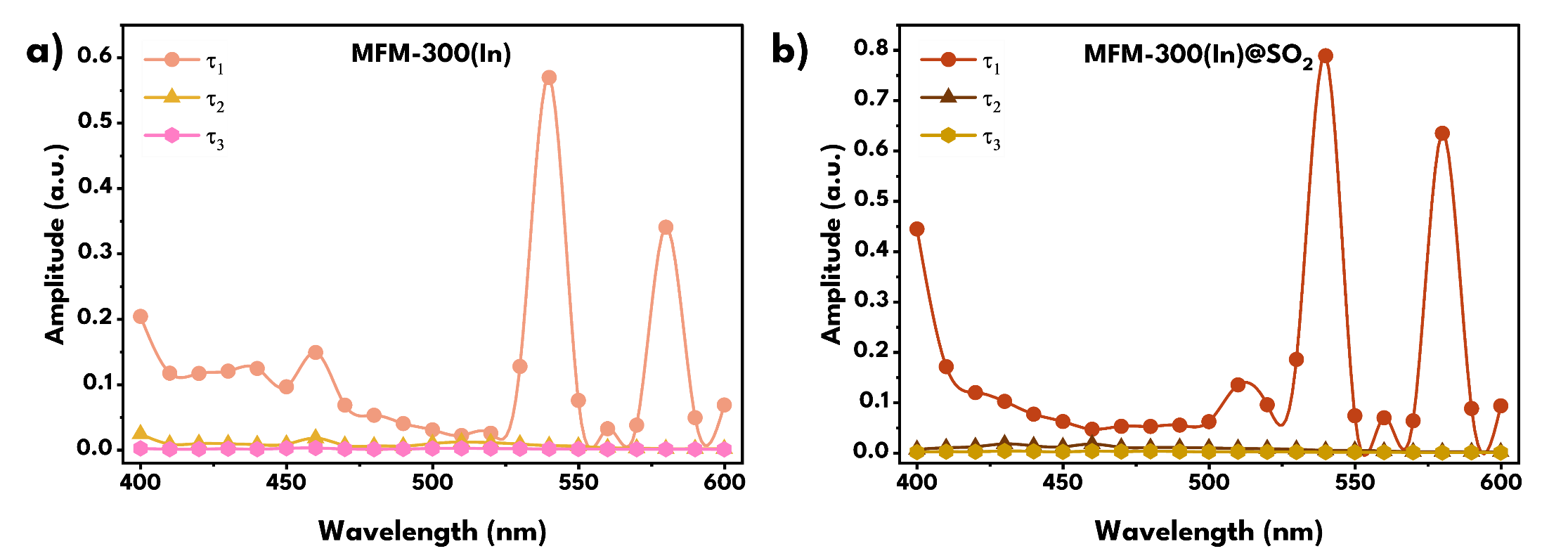


**Figure S24**. DAS of MFM-300(In) a) before and b) after SO_2_ saturation.

**S8. Determination of the limit of detection (LOD)**


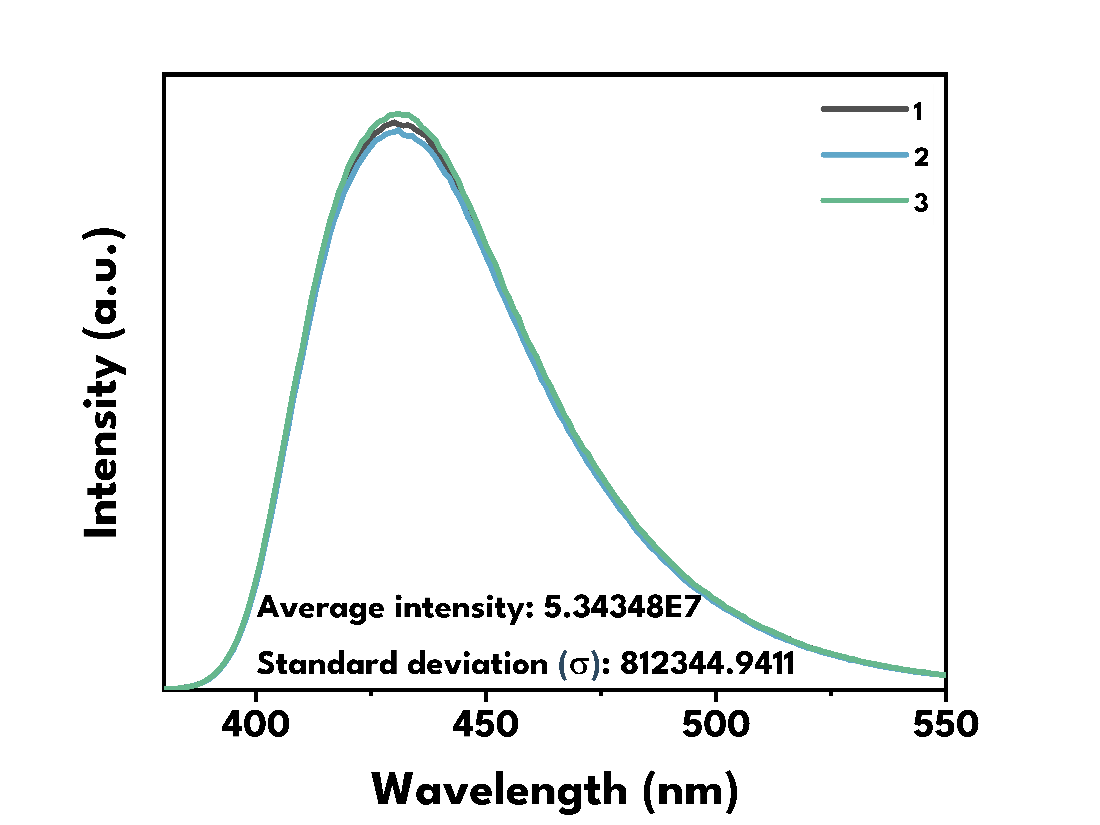


**Figure S25.** Emission spectra for three activated MFM-300(Al) materials dispersed in THF.

The limit of detection (LOD) was calculated using the following equation:^[19]^

$$LOD =\frac{3\sigma}{m} (6)$$

Where σ is the standard deviation of blank readings and m is the slope of fluorescence intensity *vs.* SO_2_ concentration plot.

The slope of the fluorescence intensity was determined by a linear fit of fluorescence intensity versus SO_2_ concentration (Figure 6c in main text). Obtaining a line equation of:

y = −47526.80x + 53185600

With a good correlation of R^2^ = 0.9926.

To obtain the standard deviation (σ) of the pristine material reading, three aliquots of a 5 mg suspension of MFM-300(Al) in 20 mL THF were taken and their emission spectra were measured (Figure S25). The standard deviation (σ) was calculated using the intensities of those 3 blank readings.

Thus, the LOD was determined with the above data:

$$Detection limit =\frac{3\sigma}{m} = -\frac{-3\left( 812344.9411 \right)}{-47526.80} = 51.2771 ppm$$

**S9. References**

1 S. Yang, J. Sun, A. J. Ramirez-Cuesta, S. K. Callear, W. I. F. David, D. P. Anderson, R. Newby, A. J. Blake, J. E. Parker, C. C. Tang, M. Schröder, *Nat. Chem.* **2012**, *4*, 887.

2 J. A. Zárate, E. Sánchez-González, D. R. Williams, E. González-Zamora, V. Martis, A. Martínez, J. Balmaseda, G. Maurin, I. A. Ibarra, *J Mater Chem A Mater* **2019**, *7*, 15580.

3 L. Briggs, R. Newby, X. Han, C. G. Morris, M. Savage, C. P. Krap, T. L. Easun, M. D. Frogley, G. Cinque, C. A. Murray, C. C. Tang, J. Sun, S. Yang, M. Schröder, *J. Mater Chem. A* **2021**, *9*, 7190.

4 M. Savage, Y. Cheng, T. L. Easun, J. E. Eyley, S. P. Argent, M. R. Warren, W. Lewis, C. Murray, C. C. Tang, M. D. Frogley, G. Cinque, J. Sun, S. Rudić, R. T. Murden, M. J. Benham, A. N. Fitch, A. J. Blake, A. J. Ramirez‐Cuesta, S. Yang, M. Schröder, *Adv. Mat.* **2016**, *28*, 8705.

5 P. H. M. Andrade, C. Volkringer, T. Loiseau, A. Tejeda, M. Hureau and A. Moissette, *Appl. Mater. Today* **2024**, *37*, 102094

6 G. Kresse, J. Furthmüller, *Phys Rev B* **1996**, *54*, 11169.

7 G. Kresse, J. Furthmüller, *Comput Mater Sci* **1996**, *6*, 15.

8 G. Kresse, J. Hafner, *Phys Rev B* **1994**, *49*, 14251.

9 G. Kresse, J. Hafner, *Phys Rev B* **1993**, *47*, 558.

10 J. P. Perdew, K. Burke, M. Ernzerhof, *Phys Rev Lett* 1997, *78*, 1396.

11 S. Grimme, *J Comput Chem* 2006, *27*, 1787.

12 J. M. Soler, E. Artacho, J. D. Gale, A. García, J. Junquera, P. Ordejón, D. Sánchez-Portal, *Journal of Physics: Condensed Matter* 2002, *14*, 2745.

13 D. B. Melrose, R. J. Stoneham, *J Phys A Math Gen* 1977, *10*, L17.

14 I. A. Ibarra, S. Yang, X. Lin, A. J. Blake, P. J. Rizkallah, H. Nowell, D. R. Allan, N. R. Champness, P. Hubberstey, M. Schröder, *Chem. Comm.* **2011**, *47*, 8304.

15 S. Yang, J. Sun, A. J. Ramirez-Cuesta, S. K. Callear, W. I. F. David, D. P. Anderson, R. Newby, A. J. Blake, J. E. Parker, C. C. Tang, M. Schröder, *Nat. Chem.* **2012**, *4*, 887.

16 L. Briggs, R. Newby, X. Han, C. G. Morris, M. Savage, C. P. Krap, T. L. Easun, M. D. Frogley, G. Cinque, C. A. Murray, C. C. Tang, J. Sun, S. Yang, M. Schröder, *J. Mater Chem. A* **2021**, *9*, 7190.

17 M. Savage, I. Da Silva, M. Johnson, J. H. Carter, R. Newby, M. Suyetin, E. Besley, P. Manuel, S. Rudić, A. N. Fitch, C. Murray, W. I. F. David, S. Yang, M. Schröder, *J. Am. Chem. Soc.* **2016**, *138*, 9119.

18 U. Noomnarm, R. M. Clegg, *Photosynth. Res.* **2009**, *101*, 181.

19 A. Sharma, D. Kim, J. H. Park, S. Rakshit, J. Seong, G. H. Jeong, O. H. Kwon, M. S. Lah, *Commun. Chem.* **2019**, *2*, 1.
